# Assembly theory and its relationship with computational complexity

**DOI:** 10.1038/s44260-025-00049-9

**Published:** 2025-09-03

**Authors:** Christopher P. Kempes, Michael Lachmann, Andrew Iannaccone, G. Matthew Fricke, M. Redwan Chowdhury, Sara I. Walker, Leroy Cronin

**Affiliations:** 1 The Santa Fe Institute, Santa Fe, NM USA; 2 BEYOND Center for Fundamental Concepts in Science, Arizona State University, Tempe, AZ USA; 3 Computer Science, University of New Mexico, Albuquerque, NM USA; 4 School of Earth and Space Exploration, Arizona State University, Tempe, AZ USA; 5 School of Chemistry, University of Glasgow, Glasgow, G12 8QQ UK

**Keywords:** Astrobiology, Origin of life, Information theory and computation

## Abstract

Assembly theory (AT) quantifies selection using the assembly equation, identifying complex objects through the assembly index, the minimal steps required to build an object from basic parts, and copy number, the observed instances of the object. These measure a quantity called Assembly, capturing causation necessary to produce abundant objects, distinguishing selection-driven complexity from random generation. Unlike computational complexity theory, which often emphasizes minimal description length via compressibility, AT explicitly focuses on the causation captured by selection as the mechanism behind complexity. We illustrate formal distinctions through mathematical examples demonstrating that the assembly index is fundamentally distinct from complexity metrics like Shannon entropy, Huffman encoding, and Lempel–Ziv–Welch compression. We provide proofs showing that the assembly index belongs to a different computational complexity class compared to these measures and compression algorithms. Additionally, we highlight AT’s unique ontological grounding as a physically measurable framework, setting it apart from abstract theoretical approaches to formalizing life that lack empirical measurement foundations.

## Introduction

Understanding evolution in the most general terms, including how the process of selection manifested before the genetic code, is of primary interest for uncovering the origin of life and the prospects of other life in the universe^1–7^. A major challenge is that current theories of evolution typically begin from some intermediate, preexisting biological system and, as such, can only describe the evolutionary dynamics within that system. Assembly theory (AT) was developed to provide a more general framework for understanding evolution and selection, allowing detection of the signatures of any evolutionary process^8^, including probing stages of evolution on Earth predating genomes. By combining a formal theoretical^8–10^ and experimental^11,12^ approach to determine whether or not observed objects are the product of evolution, AT is opening new avenues of research for life detection^13–16^, the design of synthetic life^17,18^, the design of experiments to probe the de novo origin of life in the laboratory^19^, and opportunities to look deeper into the history of life on Earth (e.g., predating the evolution of the genome)^20^. In its formalization, AT includes a new, empirically measurable form of complexity, Assembly, that adds unique features to the field of complexity metrics and opens avenues for how complexity can be measured as a physical quantity.

As with any theory that attempts to capture broad regularities in the physical world, there is necessarily a large amount of abstraction in going from measurement to theory in the development of AT^21^. This has led to some confusion about whether what the theory proposes is genuinely new^22–25^ or is already captured by other theoretical metrics of complexity developed over many decades^26,27^. Some researchers have claimed that AT is identical to several other complexity measures^22–24^, but do so by pointing to mathematical formalizations that are not formally equivalent to one another, and, as we show here, are also not formally equivalent to AT. In this work, we hope to clarify how AT differs formally and in practice from currently available complexity measures. Providing clarity to the discussion requires demonstrating formal uniqueness and understanding the connection between different theoretical concepts of complexity, and in the case of AT, the role of its ontological basis in physical measurement. The assembly index (AI), a critical component of AT, has been demonstrated to be measurable as an intrinsic physical observable in molecules. This requires being explicit about differences in the physical basis of computational complexity and AT, because the latter was explicitly developed as an empirically-grounded theory of complexity, not a purely theoretical one.

The theory of computation was not explicitly developed to solve the problem of the origin of life^28^, nor was it even devised as a formal approach to life or to physical systems. In fact, one of the central goals of theoretical computer science is formal analysis without reference to particular hardware or implementations. Nonetheless, the connection between computation and living processes has been noted by many researchers over the years^29–32^. The key ideas of theoretical computer science were laid out in the seminal work of Turing^33^ and Church^34^. Early work by von Neumann attempted to analogize the concept of universal computation to that of universal construction^35^, providing a thought experiment of an abstract machine that can construct any buildable physical object (rather than compute any computable function as a universal Turing machine does). This difference is important because one theory, that of computation, deals with the inputs and outputs of programs, how to describe these, the computational resources (time and space) required, and the complexity of algorithms for producing specific outputs. The other, that of construction, was intended to deal with objects constructed by physical processes, not data and algorithms. That is, in the latter, the constraints of physical law are necessarily foundational^36^. While von Neumann’s universal constructor was primarily a theoretical concept, it was later implemented in computational experiments with cellular automata^35^, and the distinction between computation and construction has, thus far, remained mostly abstract. Self-replicating robotics has extended this idea to the physical world, but so far requires prefabricated building blocks and are far from being universal constructors^37^. Assembly theory (AT) makes the connection of these ideas to physics and metrology^38^ concrete, thus providing the first step toward a testable theory of selection as a mechanism for constructing objects.

In what follows, we first introduce the foundations of AT as developed to date. We subsequently establish why AT is not the same as computational approaches to life. (1) We show with explicit examples how a key component of AT, the assembly index (AI), is not formally equivalent to other measures of complexity derived from computer science and information theory. Specifically, we show, with simple but rigorous mathematical counterexamples, why AI yields quantitatively different results when compared to compression algorithms like Huffman coding and Lempel–Ziv–Welch (LZW) compression, which have been argued to be identical to AI. (2) We provide formal proofs for the calculation of the assembly index of strings, showing it is not in the same computational complexity class as these compression algorithms, definitively demonstrating that no formal equivalence is possible. We demonstrate that computation of the assembly index is NP-complete, so its computation is mathematically in an equivalent class to other optimal compression algorithms. However, this similarity does not undermine its ontological differences. We make this comparison not to equate assembly theory with abstract computation, but to lay to rest certain claims made that the assembly index is equivalent to metrics and algorithms in the complexity class P, which it cannot be. We therefore (3) also discuss metrology, and how assembly theory provides a rigorous approach to the physical measurement of complexity and life detection, which has not been shown to be emulated by computational complexity measures. We discuss how empirically, the assembly index is developed as a physical observable, whereas Huffman coding, LZW compression and Kolmogorov complexity are computed from labeled data. (4) Because it is physically relevant, it should be expected that assembly, when used to detect life and evolution, will have lower false positive and false negative rates than purely algorithmic measures. Because it is a context-free approach, it is also a standardized comparison across experiments and planetary environments. Any of these points would be sufficient to distinguish AT from computational approaches to formalizing life, but we also highlight how (5) AT is not based on AI alone and reemphasize the importance of the copy number in AT. Approximating any feature of AT with a mathematical measure does not replace the physical insights the theory aims to provide or invalidate the explanations it might afford.

Any useful approach to quantifying complexity relevant to life detection (in the lab or on other worlds) must bridge the reality gap between theory and empirical testing and validation. That is, it must engage with metrology. This is a primary reason why AT was developed in the context of a modern chemistry lab’s capabilities, with its initial development as a rigorous method for laboratory studies aiming to identify molecules as biosignatures^11^. A key conjecture of AT is that “life” is the only mechanism for producing highly complex identical objects in abundance, ranging from molecules, to cells, to memes and beyond. This proposal leads to a key, testable prediction of the theory that high AI objects with a high copy number will not be found independently of evolutionary processes^39^ (see e.g., ref. ^40^ for a broad discussion on abstracting the living process). While many kinds of measures of molecular complexity computed on graphs have been developed over the years^41–43^, the key advance offered by AT was to propose and formalize a precise conjecture on the relationship between molecular complexity and life and allow an experimental test. This observation necessarily and intentionally grounds AT in experimental science, since the threshold of the copy number and AI that constitute a biosignature is an essentially empirical one. AT provides a physical measurement of how much selective constraints have been applied to generate an object and, therefore, how likely it is to be associated with a living process.

**Fig. 1:**
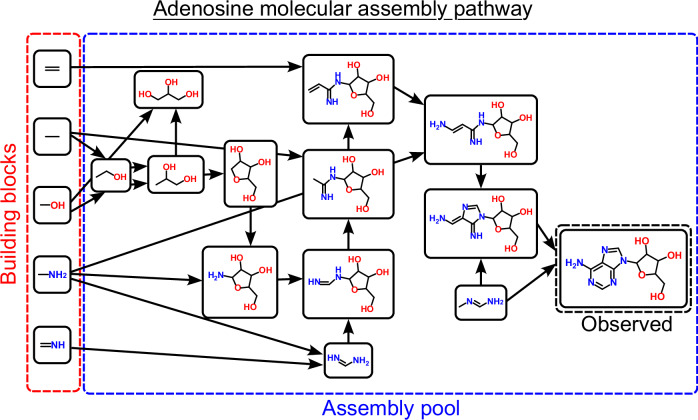
An illustration showing how a molecule can be cut into basic units, and recursively assembled from them to construct a minimum assembly path. The molecule’s molecular assembly index is the number of steps on the path.

Assembly theory has the following observables: copy number, *n*, and the assembly index, *a*_*i*_^8^. The copy number, *n*, of an object refers to how many identical objects are observed. Here, it is important to clarify what an object is in AT, because the definition departs from most definitions in physics and other fields, including as used in other approaches to formalize complexity (we shall return to this point later). In AT, objects have the properties of being finite, distinguishable, breakable (and therefore constructable), and they must be able to persist in multiple identical copies. With this definition of an object, the assembly index *a*_i_ quantifies the minimal number of recursive, compositional joining operations necessary to construct an object from its elementary parts. The exact definition of assembly index is restricted to the assembly space of physically meaningful operations, see Fig. 1 for an example of the molecule adenosine. As AT is developed, we expect it will find applications beyond the initial focus on covalent chemistry, including more abstracted spaces like minerals, gene/protein sequences, language, and many types of technology.

For molecules, both the copy number, *n* (abundance of molecular species) and *a* (minimal number of bond-making operations, allowing reuse of parts) are measurable in the lab. Assembly index *a*_*i*_, referred to as the molecular assembly index (MA) for molecules, can be measured using mass spectrometry, NMR, and infrared techniques^12^. For an observed configuration of complex matter (e.g., a system that can be broken apart to more basic building blocks), taking account of both the number of copies of distinguishable objects and their assembly indices allows quantifying Assembly, *A*, which we conjectured captures the total amount of selection required to produce it. Formally, for an ensemble of *N* total (non-unique) objects, the Assembly is defined^8^ as

1 $$A=\sum _{i}{e}^{{a}_{i}}\left(\frac{{n}_{i}-1}{N}\right)$$

where the terms of *n*_*i*_ and *a*_*i*_ are the copy number and assembly index, respectively, of the *i*th distinguishable object in the ensemble. The assembly equation, Eq. 1, represents a formalization of selection and evolution in a very large combinatorial space, the assembly space, defined as the set of recursive, physically implementable (and thus measurable) operations from which objects can be constructed (Fig. 1).

AT suggests that the combinatorial space of evolutionary objects is best understood in terms of coordinates of assembly index and copy number within the assembly space^8^. Finding objects with high assembly indices in high copy number, yields large values of *A*, implying substantial selection must have occurred^11,12^. This is because it becomes super-exponentially more difficult to produce identical copies of increasingly assembled objects with large *a*_*i*_ by chance alone (i.e., there is a super-exponential expansion of the combinatorial space for each possible joining operation). That is, observing ${n}_{i}\gg 1$ for a complex object with sufficiently high *a*_*i*_ is nontrivial, and in AT is conjectured to be impossible to happen abiotically. In AT, configurations of matter with large *A* are unreasonably unlikely to occur outside of selective processes found in what we call “life”. Here, we are using selection in a generalized sense: selection is the mechanism whereby a set of constructive circumstances yields the formation of some subsets of objects over others. This spans simple reaction rate differences to formal modern genetics, where the strength of selection would also be larger for the latter, thus yielding higher *A*. Laboratory measurements of molecular assembly indices confirm the conjecture that an observational threshold for the evidence of selection and evolution exists. To date, experimental studies of abiotic, biological, and blinded samples indicate that molecules with *a*_*i*_ > 15 are found only in living samples^11^.

## Results

### Overview of complexity measures

The ontology underlying AT as a theory of physics is different than that of the theory of computation, see Table 1. In what follows, we compare AT to computational and information-theoretic approaches to quantifying complexity. For some comparisons, we give explicit examples, while for others we do not because the differences in ontology and computability already make clear the most salient distinctions between different approaches, and in some cases these distinctions are so apparent as to make direct comparison not meaningful. For establishing mathematical uniqueness, we take the simplest and most illustrative approach, in the form of basic counterexamples. We consider cases where the assembly index is distinct for two outputs, but those two outputs yield the same value in another complexity measure; and we show cases where the assembly index is the same for two outputs, but other complexity measures yield distinct values. Either scenario would be enough to show that the assembly index is not one-to-one with the comparison complexity measure.

**Table 1:** Comparative ontology of complexity measures

Complexity Theory	Operands	Measures	Description/Details
Algorithmic complexity	Classes of mathematical problems	Computational complexity Kolmogorov complexity;	Measures are concerned with optimal (minimal) descriptions of products of computational procedures, or the computational resources required for an algorithm that could produce the given output
Compression and statistical complexity	Data and its representations	Huffman code; Lempel–Ziv–Welch compression; Information theory	Measures provide data transformations to generate reduced representations
Physical complexity	States of physical systems	Logical depth; Thermodynamic depth;	Measures are concerned with the physical resource needed to compute a minimum description, or the probabilities of observed paths to produce a state
Assembly theory	Physical objects	Assembly index	Measure is concerned with the minimum construction as an intrinsic and measurable physical attribute

Examples of different kinds of complexity theories and measures, describing their operands, measures, and conceptual underpinnings.

Before we dive into computational complexity, it is important to note that there are different notions of equivalence. Computational complexity employs a weak notion of equivalence that does not require a strict one-to-one equality for metrics in the same broad complexity class. Physics and the natural sciences often require a stronger notion of equivalence associated with equality and one-to-one correspondence, often rooted in mechanism, and this notion is what we would require for the assembly index. These different disciplinary notions of equivalence have caused a lot of confusion.

The most widely discussed measure of computational complexity is the Kolmogorov–Chaitin complexity^44,45^, also known as algorithmic complexity. Formally, the algorithmic complexity *K(x)* of a string *x* is defined as the length of the shortest computer program, which, when run, will output *x* (assuming a fixed programming language). That is, *K*(*x*) is the length of the minimum description of *x*, found by searching over all programs that output *x* in the language *x* is described in. The value of *K*(*x*) for a given string, therefore, depends on the choice of description language (and, for example, might be different if we wrote the program in *C* versus Python)^46^. Foundational results in the theory of computation establish there can be no algorithm that determines whether an arbitrary program will produce a given output as this would involve, at least, deciding whether the program will halt, which is uncomputable^33^ and therefore *K(x)* is also uncomputable^44,45^. For the example above, the two strings have equivalent size, but their complexity is not the same: $K\left({x}_{1}\right) > K\left({x}_{2}\right)$, and *x*_*2*_ would thus be described as more compressible. However, there is no way to guarantee *x*_*1*_ does not have some hidden pattern in it that makes it just as compressible as *x*_*2*_ (that is, although we have shown $K\left({x}_{1}\right) > K\left({x}_{2}\right)$ for the two shortest programs we were able to identify, it could still be possible that there exists an algorithm K’$\left({x}_{1}\right)\le K\left({x}_{2}\right)$).

In Abrahão et al.^22^ and Ozelim et al.^24^, the authors make an argument that since AT does not offer any innovations in approximating *K*, it is rendered redundant by preexisting methods of lossless compression. However, this represents a fundamental misunderstanding because approximating *K* is not, and was never, the goal of AT. It is our view that *K* is not as relevant as a physical measurement of complexity generated by evolutionary processes. The assembly index comes to estimate a lower bound on the chance of the random generation of the molecule from molecular inflow into the system, by formalizing the minimum causation necessary to make an observed configuration. The algorithmic complexity, on the other hand, is the minimal size of the program required to generate a certain output— given a universal turing machine. The latter introduces a deep philosophical challenge in that the construction of the molecule requires construction of the molecule itself by the UTM (we ignore for a second the fact that a UTM cannot construct molecules), but also the construction of the UTM itself (this in essence was von Neumann’s point in developing the idea of a universal constructor). Construction of such a reliable computer will require many more steps than the construction of the molecules that we are interested in when detecting life. Though it is true that very simple physical systems can exhibit universal computation when properly encoded by human intervention^47^, there is no evidence of any non-evolved system that exhibits universal computation, that can reliably control molecular reactions, and can form itself within a small number of steps in finite time. In fact, the only process that we know of anywhere in the universe that can build such a machine is the evolution of life itself. Until we can find such a process, or a reason for it to exist outside of evolution, the assumption that before the origin of life, molecules form in large numbers only through assembly processes in the absence of computers seems well-founded. Thus, the assembly index does not come to approximate *K*, instead*K* is a poor approximation for the minimal chance of formation of molecules, a task that the assembly index was specifically designed for. As an example, let us look at a polymer that represents the first 100 digits of the number *e* in some base, that is present in an abundance such that it can be unambiguously detected. This is a relatively simple computation, and therefore, the program to compute it can be small. However, the length of this program will not represent the chance for the formation of such a molecule. Instead, it will form by assembly of the monomers plus assembly of the 100 digits, very similar to the assembly index of any other random polymer of length 100. Without a universal Turing machine, the fact that there is a small program to generate the sequence is meaningless, and the hard task in the evolution of life is building the machines in the first place.

### Motivation for AT and the Assembly index

Assembly index was developed as an exact, objective measure that can be probed by different experimental techniques, capturing symmetries and reuse of fragments in the assembly pathway to construct a molecule (Fig. 1). This distinguishes it from statistical notions of complexity, which rely on computed probabilities from the observed frequency of reuse of segments in a data structure. Nonetheless, the assembly index has been critiqued as both a restricted version of Huffman coding^22,23^ and described as reducible to Shannon entropy^24^. Formally, a “restricted version” or “reducible” should imply that one formulation is a formal subset of another. For Huffman coding, Shannon entropy and assembly index, it is easy to explicitly show these are not equivalent and therefore that assembly index is by no formal means a “restricted version” of Huffman coding or reducible to Shannon entropy.

Information theory provides the theoretical underpinnings for efficient data compression and coding techniques, establishing limits on to what degree data can be compressed without losing information (lossless compression), and how information can be transmitted over a noisy channel reliably. Concepts like algorithmic complexity^44,45^, Huffman coding^48^, arithmetic coding^49^, and channel capacity^50^ are all grounded in information-theoretic principles. Information-theoretic measures like Shannon entropy^50^, mutual information^51^, transfer entropy^52^, and synergistic information^53^, etc., are widely used to characterize the complexity, dependencies, and similarities in data across complex systems^54,55^.

Shannon entropy is a powerful tool due its generality, but this also means one must exercise caution in its interpretation as it can be applied to any data labeling scheme one might wish to define^56,57^. Indeed, Shannon entropy provides a good example of how being explicit about the ontology of the abstract variables that appear in our different mathematical concepts of information is critical to understand their utility (see Table 1). For example, if *X* labels the microstate of a physical system, the mathematical form for Shannon entropy is nearly identical to the thermodynamic entropy^58^ $S\left(X\right)={kTH}\left(X\right)$ where here *k* is the Boltzmann constant and *T* the temperature of the system. This allows a physical interpretation that has utility: there are many reasons to think that many complex systems, from languages to computer algorithms, would target distributions of states, and the energetic costs of moving between states, as a key feature for refinement. The formal equivalence of Boltzmann and Shannon entropies has therefore led to interesting work on the connections between information theory, computation, and thermodynamics^59^. Stemming from the work of Landauer^60^, much of this effort has focused on the minimal energy required to perform a computation by casting computations as entropy transformations, and on showing that storing and erasing information comes with certain energetic costs, which includes experimental verification^61^. Such connections have even been used to bound the thermodynamic efficiency of the ribosome as a string writing device.

**Fig. 2:**
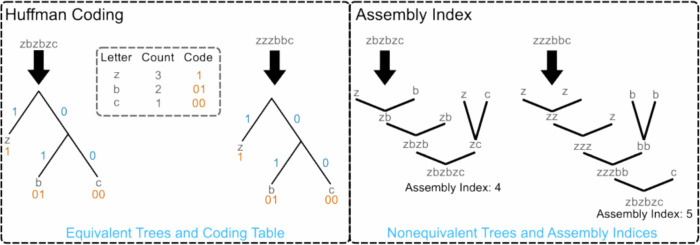
Two strings with different assembly index but identical Huffman trees. A single case such as this is sufficient to demonstrate that the assembly index cannot be a “restricted case” of Huffman coding, because it demonstrates that the assembly indices cannot be mapped one-to-one with Huffman trees.

Huffman coding^48^ is among the most widely used lossless data compression algorithms. A Huffman code is constructed by first building a frequency table of characters and their occurrence counts, see Fig. 2. The compression efficacy of Huffman encoding depends on the character frequency skew, where data with highly frequent characters are compressed the most, and data with uniform distributions of characters are compressed the least.

As a simple illustrative example of formal differences between assembly index, Shannon entropy and Huffman coding, we consider the strings:

$$\begin{array}{l}zbzbzca=a\,of\,4\\ zzzbbca=a\,of\,5\end{array}$$

These strings are identical in length and composition (with the same number of *z*’s, *b*’s, and *c*’s), and thus have the same string entropy and Huffman coding. However, the two strings have different assembly indices (4 and 5, respectively).

The Huffman tree is constructed by creating a node for each character and repeatedly combining the two least frequent nodes/characters into a new parent node, until only one parent node, the root node, remains, see Fig. 2. For the example above, the tree that generates the code alphabet is identical for the two strings (because they have the same character frequencies) with the following code assignments

$$\begin{array}{l}z\to 1\\ b\to 01\\ c\to 00\end{array}$$

which results in the encoded output strings

$$\begin{array}{l}zbzbzc\to 101101100\\ zzzbbc\to 111010100\end{array}$$

There are several observations of note here. Two strings with different assembly indices can produce identical Huffman trees and code alphabets. The two representations, Huffmann coding and assembly index, are not one-to-one because the Huffman trees are identical for strings that have different assembly spaces, see Fig. 2, providing a counterexample proof that AT cannot be a subset of Huffman coding (contrary to the claims of ref. ^22^) because there can be no 1:1 map between the two applicable in every case. This establishes that the assembly index is formally unique when compared to Huffman coding.

Assembly index is not a statistical measure and does not rely on the frequencies of characters; instead, it aims to capture features of the recursive composition of objects as they are constructed from a base alphabet. By contrast, Huffman trees are often highly degenerate because they map the specific structure of strings onto character frequencies, which is a many-to-one mapping. In general, the assembly index is more weakly dependent on character frequencies than such statistical measures, and it will yield different values in many cases where Shannon entropy or Huffman coding would not resolve differences between strings.

Some have argued that lossless compression, like that involved in generating a Huffman code, is relevant to evolution, particularly in describing genomes^62^. However, genomes are highly constrained by the evolutionary history of the ribosome, and physical constraints on protein folding^63^. As such, compression might not yield the most accurate insights into evolutionary refinement of genomic architecture, especially as it does not account for the physical and physiological constraints present in all cellular life^64–66^ (or viruses^67^). Given that compression-based measures are sensitive to the choice of data representation, they must be applied with care in a biological context: the labels humans use for data to describe living things may not be the same labeling scheme those organisms implement themselves, even if it were the case where an organism was indeed performing data compression.

By contrast, the aim of AT is different because it does not presuppose that a compressed description is necessary, and the theory is agnostic to assumptions that cellular life is doing any kind of computation or algorithmic compression. That is, AT does not rely on a human or computer-based labeling scheme, or any kind of statistical description of data; instead, it aims to identify the underlying structure of the physical space in which selection operates (the assembly space).

**Fig. 3:**
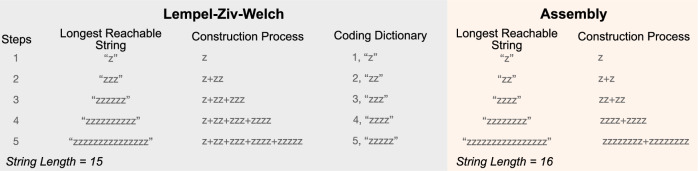
The most compressible string of a single letter as represented in *n* steps of Lempel–Ziv–Welch (LZW) compared with *n* assembly steps, demonstrating that the scaling of the two algorithms is not equivalent and by extension there can be no formal equivalence between the two.

The assembly index has also been criticized by the same group of researchers as identical to other compression algorithms, including the Lempel–Ziv compression scheme^22–24^. But again, we can establish formal independence with basic worked examples for strings. We consider Lempel–Ziv–Welch (LZW) for an explicit example^68^. For LZW a string sequence is read from left to right, and a lookup table is built for new substrings. The longest matching substring is chosen, and a new substring which includes that substring and the next letter in the input is added to the lookup table along with a new index. Regular signals can be characterized by a small number of patterns and hence have low LZW complexity. Irregular signals require long dictionaries and hence have a high LZW complexity (most schemes employ a maximum dictionary size based on the number of available character bits). The easiest way to establish that LZW is not formally equivalent to the assembly index is to look at the scaling of the simplest string case, a repeated single letter (Fig. 3). If we use LZW compression, string length, *l*, will grow following:

$$\begin{array}{l}{l}_{i+1}\,={l}_{i}+i+1\\\quad {l}_{1}=1\end{array}$$

where *i* counts the number of compression steps to build up to the next most compressible string of a single letter. This yields a scaling of the longest string length that can be reached by *n* compression steps (or dictionary construction steps) as

$${l}_{n}=\left(\frac{n\left(n+1\right)}{2}\right)$$

Assembly index looks for the shortest path using parts that have already been constructed, and it has previously been shown that this string will grow with assembly steps like^8–10^:

$${l}_{a}={2}^{a}$$

where we have used the relation of assembly index to string length for single character strings, *a* = *n*–1, for *n* assembly steps. It is clear that the scaling of length versus steps for assembly index and LZW are not equivalent: for *n* steps in LZW compression, string length scales like *l*_*n*_ $\propto$ *n*^*2*^ (in the limit of large strings) compared to the much faster scaling of ${l}_{a}\propto {2}^{a}$ for *a* + 1 steps along an assembly path. Inverting these we find the number of compression steps scales with string length as $n\propto \sqrt{l}$ for LZW schemes, and the number of assembly steps scales like $a\propto {\log }_{2}l.$

For LZW compression to be implementable, it requires the development of a rather sophisticated structure of reading, labeling, and storing data^68^. It is not clear how many complex systems will emergently discover such sophisticated algorithms for data processing. It also depends on how sequences of data are read: data with similar character count, repetition of structure etc., can have very different LZW compressions because these are highly dependent on reading the data from left to right, what characters are at the start of the sequence, and therefore are sensitive to how one chooses to read the data^69^.

### Comparison of AT with other complexity measures

Other LZ algorithms such as LZ77 have also been claimed by the same group of authors to be equivalent to, or outperform, assembly index^22–24^. However, these algorithms are always constructed to read data with directionality (e.g., from left to right), with no look-ahead. This feature makes these algorithms efficient—they do not need many passes over the file, or often just one. The Assembly index, on the other hand, looks at global optima. It does not just do a look-ahead; it goes much further and looks over all possible assembly pathways. This is because there is no requirement for speed, but instead to reach an inherent, global, property of the molecule. Sacrificing this feature to make LZ algorithms look more like assembly index calculations therefore removes a central feature of LZ compression schemes, making the direct comparison of these algorithms more of a mathematical exercise in approximating assembly index with a LZ like scheme, then a meaningful statement of exact equivalences between LZ algorithms and assembly index. In the field of computational complexity measures, differences of this degree are sufficient to categorize algorithms as distinct. Indeed, as we will show in the next section, computation of the mathematical metric of the assembly index is NP-complete, which puts it in a different computational complexity class than most compression measures, including LZ, meaning it must be formally distinct from them. We note that for molecules, algorithms to compute exact values of assembly index are nontrivial^70^, and we do not expect any of the approximate approaches presented in ref. ^22^ to work in many important cases (this again goes back to the issues of representation of data and labeling, the algorithms the authors implement in ref. ^22^ will not yield the same results as assembly index in all cases, and would require modification for each new data type for encoding molecular data). The mathematical relationships of assembly index to optimal compression are indeed intriguing, but it is important to keep in mind the difference between math and physics, and the assembly index is intended to be a mathematical representation of a physical attribute of evolved objects.

### Computational complexity class of AT

In theoretical computer science, a common way to classify algorithms is by their computational complexity class^27^. If two algorithms are in the same complexity class, they could be equivalent in the difficulty of their computation, although the form of the metrics may not be one-to-one or indicate the same things about the outputs. However, if two algorithms are found to not be in the same complexity class, it is understood they will never be shown to be formally equivalent (assuming P does not equal NP). In the supplement, we show that the formal definition of assembly spaces, as originally stated by ref. ^11^ and expanded on in the supplement, is NP-complete. It should be noted that, thus far in this paper, we have focused on strings since they provide simple examples, and while they have not so far been the primary focus of the developers of AT, these are the focus of several works that are critical of the theory. Indeed, AT is more general than the string structures discussed in these criticisms. AT was developed to deal with molecules, and these are much more complicated structures with more complicated attachment rules than strings; thus, we would not expect all properties of string assembly to generalize to the more complex case of molecules. AT, and within it the calculation of the assembly index, covers a wide range of complexity classes. In the proof in the supplement, we give an exact definition of assembly index and show that a set of string assembly problems are NP-complete. The problem of tiling—whether a shape can be fully covered using a set of tiles—can be included in assembly space and is very similar to assembling a molecule using a set of input molecular structures. Tiling of 2D and 3D shapes is known to be NP-complete^71^. Answering whether a molecule can be assembled out of a set of tiles is simpler than answering what the minimal number of steps to do so; therefore, we know that molecular assembly must be at least NP-complete. Other assembly problems could be simpler. Thus, string assembly is probably NP-hard, whereas the problem of assembling strings of different lengths out of a single letter is equivalent to the shortest addition chain problem, whose exact complexity is unknown, but possibly lower^72^.

It is significant to our discussion that the assembly index is NP-complete because most compression problems can be solved in polynomial time. Denoted P, this class is defined as the space of all algorithms that can be computed in time bounded by a polynomial function of the size of the input. This makes sense for compression algorithms, because the goal of compression is providing data representations that are easy to compute. Another widely studied class of algorithms lives in the class of non-deterministic polynomial time problems, denoted NP, defined as the space of algorithms where a known output has a proof that can be verified in polynomial time by a deterministic Turing machine, or solved in polynomial time by a non-deterministic one. Non-deterministic Turing machines can be simulated by deterministic Turing machines, but this simulation generally requires exponential time in the worst case. After that, the next class of interest is non-deterministic polynomial time complete problems, denoted NP-complete. NP-complete problems form an equivalence class, defined as the space of algorithms where it is possible to reduce one NP problem to another NP-complete problem in polynomial time. This is relevant because the class of NP-complete problems cannot be mapped to problems in P. Problems in P, however, can be trivially mapped to problems in the NP-complete class, since the problems in P are an easier subset of the NP-complete problems.

The result that computing the assembly index for strings is NP-complete has several important consequences. First, NP-complete problems are expected to have an exponential worst-case runtime^27^, and so algorithms that find the assembly index could have an exponential runtime. Second, proving assembly index is NP-complete means it cannot be strictly equivalent to LZ algorithms, Huffmann coding, or Shannon entropy because these are all in the complexity class P and computing these scales like *O*(*n*)*log*(*n*), *O*(*n*), and *O*(*n*) respectively^73,74^ as opposed to *exp*(*n*). Indeed, any complexity measure that lives in P cannot be strictly equivalent to the assembly index, which is NP-complete. There will be NP-complete problem instances that cannot be mapped to problem instances in P. This result implies our counterexamples are not just specific cases but represent general and fundamental differences between the assembly index and these compression algorithms. Comparisons to Huffman coding, Shannon entropy, LZW, and LZ77 can only be approximate comparisons to assembly index and will never represent exact equivalences.

### Purpose of AT and assembly index

A critical divergence between these compression algorithms and AT lies in the interest in optimality. Compression algorithms, while theoretically concerned with finding the shortest possible representation, are more often used in a context where one settles for “good enough” solutions that achieve practical compression ratios quickly. The actual global minimum is not essential for their primary function of efficient data reduction. However, in AT, the global minimum (assembly index) is of central interest because it captures minimum causation as an intrinsic physical feature. Note we have not ruled out that computation of the assembly index is equivalent to some NP-complete compression algorithms; for example, optimal, i.e., minimal output length, text-substitution, and run-count compression algorithms are NP-complete^74,75^. Shannon entropy is in *O*(*n*) where *n* is the length of the input string, since the algorithm only must iterate over the string once to calculate the frequency of each character. Compression algorithms in the computational complexity class P, such as those reviewed herein, are useful because they are efficient, and in some cases, can be computed with a single pass over the input. Compression algorithms in the NP-complete computation class are intractable for compression, and therefore, efficient heuristic compression is universally preferred. Assembly index might be considered an optimal compression strategy in this sense since it returns the minimum object reconstruction path, but it is important to recognize that the goal of AT is not to provide a formal measure of compression. One should not confuse the mathematical representation of a theory with what it describes about the physical world, and indeed, many mathematical theories of nature can be expressed by equivalent but different representations simply due to the nature of mathematical description. A trivial example is how many mechanistic derivations will lead to a square root of time scaling, but not all of these are a random walk. The assembly index, as far as what it is meant to represent about the physical world, does not describe algorithmic compression, even if its formal representation may be equivalent mathematically to descriptions of some optimal compression algorithms. Finding a formal equivalence with another algorithm that is likewise NP-complete would in no way invalidate the theory, but could be useful in providing other ways to compute the assembly index, which might in turn be useful in testing AT’s theoretical predictions against experimental data. Compression algorithms used in practice are heuristics that attempt to approximate optimal compression while being computationally tractable. Similarly, AI requires heuristic approximations for large problems. Heuristics often leverage assumptions about the problem domain to improve their approximations. In the domain of string AI, compression heuristics such as LZ compression may indeed provide useful insights. However, in the case of approximating molecular assembly, relevant to agnostic biosignature detection, the best heuristics will likely leverage domain chemistry knowledge.

Many complexity measures can be correlated while not being formally identical. In fact, it should be expected that most computational complexity measures will be correlated with output size, and indeed, all measures we have discussed herein are (at least weakly) correlated for this reason. In Ozelim et al.^24^, the authors claimed correlations between assembly index and other complexity metrics invalidate the use of assembly index as a physical observable. This represents a misconception about the differences between correlation and metrology. It may be possible to correlate all sorts of variables, but only the assembly index has been shown to be reconstructable from the physical properties of molecules as probed by mass spectrometry, NMR, and infrared^11,12^. It is worth pointing out that the assembly index was constructed exactly because the physical size of a molecule does not say much about its complexity. There are many large molecules, e.g., polymers, that easily occur in a chemical sample that are large and can form in the absence of evolution because they are composed of repeating units or other molecular symmetries. Therefore, size and all complexity measures whose only empirical measurement is correlation size are unreliable methods for life detection.

It is widely recognized that correlation neither implies causation nor equivalence. As a simple illustrative example of the category error of confusing correlation with mechanism, consider the two physical laws mathematically represented by the equations $\frac{{T}^{2}}{{r}^{3}}=\frac{4{\pi }^{2}}{{Gm}}$ and $E=m{c}^{2}$. Both are functions of mass with one effective parameter, but each calculates a different physical quantity and in a different context. They are built on different mechanisms, one describes planetary motion and the other gives the energy of a particle in its rest frame as derived from special relativity. Superficially, the two correlate perfectly because of their linear dependence on *m*, but this in no way implies the two theories are equivalent or capture the same physics. This is an absurd example aimed at highlighting that trivial correlations are possible even for well-developed theories. Likewise, correlation in complexity measures, especially when controlled by overall output size, does not imply equivalence. For example, many efforts approximate Kolmogorov complexity, which is formally uncomputable, with Lempel–Ziv, or other compression schemes, which are in the class P. This means that Lempel–Ziv will fail at the most interesting cases of Kolmogorov complexity. Furthermore, the point of building measures and theories in physics is to compactly capture mechanism, and so the other complexity measures that do not share the same mechanistic foundations should not be assumed equivalent because of correlations. There is a long history in the physics of measurement about the challenges of developing measurement schemes that accurately capture physics, see e.g., Chang^38^ for a history of the measurement of temperature.

In Marshall et al.^11^, assembly index was shown to be reconstructed by counting the number of MS/MS peaks, demonstrating a meaningful measurement of assembly index using mass spectrometry. Fragmentation of molecules via mass spectrometry is expected by the theory to reconstruct the structure of the assembly space for molecules via recursive decomposition of molecular fragments. Thus, the measurement using MS/MS maps directly to the ontology of the theory, rendering the correspondence of assembly index and MS/MS peaks physically meaningful. Subsequently, inference of molecular assembly index was shown to lead to convergent results for mass spectrometry, NMR and infrared measurements, again considering molecules as physical objects in AT, where these techniques reconstruct features of the assembly space^12^. This means that the assembly index is invariant with respect to the measurement technique used to read it out, motivating the interpretation that the molecular assembly index is an intrinsic molecular property of a molecule.

The authors in Uthamacumaran et al.^23^ and Ozelim et al.^24^ show a correlation of different compression measures to assembly index and mass spectrometry data, but do so devoid of ontology and without an explanation for why their reported correlations should be metrologically meaningful. Specifically, they show how the length of string-based representations of molecules (e.g., using SMILES) and compression values for these strings are correlated with the MS/MS peaks in molecular data^24^. These correlations have no basis in chemistry, but they use these kinds of correlations to argue for why the assembly index should not be considered as measurable or any better than any other complexity metric that could be used in a similar way^23^. However, their results should be trivially expected based on the scaling cross-correlation of complexity measures with output size alone, and this does not recover what assembly index is capturing. A simple example is to consider oligomers, which by Ozelim et al.’s analyses would have values that scale with their size, but the assembly index would yield much lower values due to recursion in the use of monomer fragments (this example is along similar lines to the scaling differences noted above in our discussion on the differences between assembly index and LZW). It is well documented that assembly index has a weak correlation with molecular weight, which is progressively weaker with increasing molecular weight (e.g. see Fig. 2b in Marshall et al.^11^ and Figure 5a in Jirasek et al.^12^ where it is shown that the upper limit of assembly index scales roughly with molecular weight and the lower limit with the logarithm of the molecular weight). The correlations in Ozelim et al.^24^ with the size of the molecule do not recover what the assembly index is capturing.

**Fig. 4:**
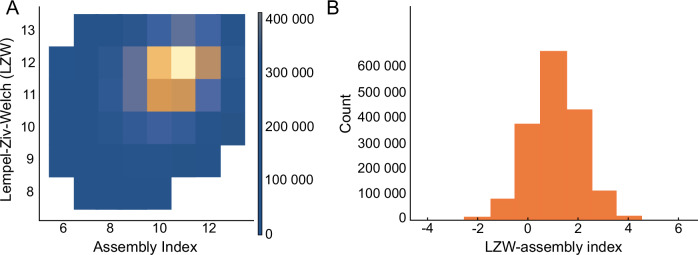
Comparison of the assembly index, a, and the Lempel–Ziv–Welch compression for every permutation of “aaaaaabbbbbbcccc”. **A** The density map of a compared with LZW, where the two have a weak Pearson correlation of 0.25. **B** The histogram of the difference between a and the length of LZW (given as LZW –assembly index) showing an approximate Gaussian distribution. These results confirm that when controlling for size, assembly index and LZW compression are weakly correlated.

In general, if one is not careful to control for string length, correlations across complexity measures will be artificially inflated. This is the kind of spurious correlation we pointed to in the example of mass above, where correlations are apparent but only due to superficial similarity. Some correlation is evidenced in ref. ^24^, where the authors show a plot of correlation between LZW and AI by plotting both scaling with size (Fig. 2 in ref. ^24^). Such correlations are much weaker if one does account for size and would get weaker with larger objects because the assembly index’s correlation weakens with object size. As an explicit example, we consider LZW compression and assembly index, comparing the ratio of the two values for strings of fixed length and composition. We considered 10,000 random rearrangements of the string “aaaaaabbbbbbcccc” and took the ratio of assembly index to the number of bytes in the compressed string as computed using LZW, see Fig. 4. We find that this ratio roughly follows a Gaussian distribution, showing assembly index and LZW are only weakly correlated with each other when controlling for size. The Pearson correlation between the two is very weak at 0.25. The set of permutations of “aaaaaabbbbbbcccc” is a small subset of the strings of length 16 constructed from the characters {a,b,c}. We have already shown for the simplest case of strings composed of a single character how LZW scales with length *l* like $n\propto \sqrt{l}$ while the assembly index scales like $a\propto {\log }_{2}l$. This implies that the worst mismatch between LZW and the assembly index will grow at least as fast as $\sqrt{l}/{\log }_{2}l$, approximated as $\sqrt{l}$ for large *l*: that is, while both correlate with size, the worst mismatch between the two increases with size. This is also evident in ref. ^24^, where the scatter correlating assembly index and LZW increases with string length. Given that the assembly index is constructed from the measurement scheme itself, its correlation is not due to averages over size-correlated effects, which give a superficial appearance of similarity even though cases of significant interest, particularly those for biosignature science, should be expected to yield very different results.

Compression algorithms will assign values to molecules that depend on the choice of representation of the data. Theoretical physics proceeds by using specific language in which all statements are intended to carry a physical meaning. In AT, this is done by selecting a measurement scheme for system representation and specifying a set of operations that correspond to physically realizable transformations. For molecular AT, this choice is well-motivated by knowledge of covalent chemistry^76^. The frontier of the theory is in developing metrology for new domains of application, where work is to be done to ground equally well-motivated choices for construction of the assembly space in empirical measurement^77^. Computational complexity methods face serious ambiguities as tools for physics because they suffer from an arbitrary choice of universal computer, whereas AT resolves this ambiguity through metrology, constructing the assembly space from laboratory-based measurement.

The measures the authors of refs. ^22–24^ introduce all suffer from ambiguity in the choice of data representation, analogous to the ambiguity in choosing a universal computer for *K* (see above). They will lack an absolute ordering for complexity that would accurately delineate the boundary between non-life and life, obfuscating the distinction that AT formalizes. Indeed, different data representations of the same molecule, e.g., in the form of SMILES^78^, or INCHI^79^, graph-based representations, or even mass spectrometry data files, should be expected to yield quantitatively different results for the computed compressibility of the molecular data. Thus, a general correlative trend might be established, but the values assigned to specific molecules will depend on how one chooses to represent the data (e.g., this characterizes what is observed Fig. 7 in ref. ^24^). Using these correlations would provide a very poor basis for life detection because it should be expected that the same molecule will fall in different locations on such correlation plots depending on choices made representing the molecular data. That is, we should expect weak correlation to be possible, but the structure underlying where specific molecules lie to be representation dependent. This is untenable for life detection, because as we have already pointed out, one could choose any representation of the data and its compression that would represent any claim one might wish to make about the relative complexity of the molecules (Section 1.1), thus rendering the correlations useless without an underlying ontology forcing a choice of representation, which is a key difference between physics and computer science.

Many considerations about the physical nature of the process of computation, as well as the physical instantiation of outputs of algorithmic procedures, are not accounted for by the theory of computation^27^. This has motivated several researchers to develop related complexity measures that might capture more physically relevant details. We now discuss these measures, and why AT represents a significant advance in the development of a physics of complexity, measurable in the laboratory. Originally developed by Bennett^80^, logical depth was inspired by universal computation, but with a goal to quantify achieved complexity rather than potential complexity. Logical depth is distinct from algorithmic complexity because it quantifies complexity based on the work a computer must do to produce a given output, rather than an abstracted notion of program size. Formally, the logical depth of a string is defined as the execution time, or number of machine cycles, it takes a computer to calculate the output from the shortest possible program. In general, these yield different results: random objects have little depth despite having potentially long descriptions, while structured objects like repeating strings are expected to have a depth more coincident with the difficulty of the process that generated them. Logical depth has an advantage in applications where it is necessary to distinguish complexity due to randomness as distinct from meaningful computational work. However, like algorithmic complexity, it is dependent on the choice of language to describe the output, and in general, the shortest program is not computable.

Thermodynamic depth was introduced by Lloyd and Pagels as a measure of macroscopic complexity^81^ and is closely related to, but differs from, logical depth. Thermodynamic depth captures the number of steps a system passes through to reach the current state. The goal of thermodynamic depth is to quantify complexity as a physical property of a state, noting that complexity should be a function of the process, or assembly routine, that brought the state into existence (and not just a description of the state)^38^. We have now arrived at the first comparison complexity measure reviewed herein, which deals explicitly with physical states rather than computational programs and their outputs. Thus, this is the first candidate, besides the assembly index, that is explicitly about physics and what can be experimentally determined. Lloyd and Pagels tried to empirically ground their measure of physical complexity by defining it as a continuous function of the probabilities of the experimentally defined trajectories that can result in a macrostate, *d*, which might be observed in a lab. The thermodynamic depth is thus defined as $D\left(d\right)=-{kln}\left({p}_{i}\right)$, where *p*_*i*_ is the probability, the system arrived at state *d* by the *i*th observed trajectory and *k* is an arbitrary constant. Thermodynamic depth will depend on what experiment is done, e.g., via empirically observed frequencies, *p*_*i*_, of the observed paths. Its value can therefore differ for the same macrostate simply because the experiment was prepared differently (or more experiments were done). This introduces subjectivity in its definition^82^.

In a chemical system, the thermodynamic depth would depend on the probability of the reaction pathways that produced it^81^. This is untenable as a complexity metric for life detection because the same molecules will have a different thermodynamic depth in different contexts (where the reactions to generate the molecule would differ). This measure is therefore not standardizable across different planetary environments or different experiments. Also, one cannot infer thermodynamic depth from an observation of a complex chemical system at just one point in time, as one needs to know the full history of its formation: this information is almost never available for many scientific questions of interest. These challenges are exacerbated by the astronomical size of chemical space, where counting all possible reactions, pathways, and molecules is not computationally possible^83–85^. The assembly index, by contrast, is independent of the path that produced the object(s), rather it depends only on the minimum path that could have produced the object, and therefore can be measured without knowing formation history. It will yield the same value for the same physical object(s) anywhere in the universe we might observe them. The length of the minimum recursively constructed path to make an object is a unique value for every object (even in cases where there may be degeneracy in minimum paths for the object’s construction, the minimum path size is still a measurable value).

Falsification of any physical or chemical theory should come from experimental and observational tests of the explanations it provides, and the predictions it makes. AT was developed by critically addressing the question of how complex a detectable molecule must be before we know it was not formed by a random or undirected process. Exploring this idea in a laboratory setting allowed the concept of causal or contingent control, at the molecular level, to be formalized as the explanation of how highly complex objects are produced in abundance. AT, as a theory, is still in its infancy, with many open questions remaining about the mechanism for selection, and the transition in Assembly from the abiotic world to the biotic world. What is clear is that AT is poised to help uncover how selection, as a driving force, allowed the evolution of evolution in a manner that is testable with standardized measurements. This is because AT provides a measure that can detect the emergence of evolution in chemical experiments, as well as giving a new approach on which the emergence of evolution can be theoretically studied. As the theory develops, it will be important to explore its foundations. In this regard, it is worth noting that any good scientific theory should be “hard to vary”^86^, especially as concrete predictions are made that can be experimentally or conceptually tested and built on. This is one reason the assembly index having an exact value, not dependent on data representation, is critical in its role as a foundation of the theoretical framework of AT.

Critical to the conceptual shift presented by AT is the central role of copy number, which so far, we have not addressed here. Criticisms of AT as redundant or not new, have not addressed the bulk of the theory, including the structure of the assembly equation and its role in formalizing selection and the observed assembly space as a necessarily contingent structure^22–25,87^. In AT, objects must be found in multiple copies. This means that in applications of the theory, it is not meaningful to talk about the assembly index of objects independent of any considerations of how to measure and count identical copies of them (where “identical” is defined within measurement uncertainty). Whilst this may seem an ad hoc requirement, it is a critical feature of AT’s ability to uniquely identify objects as products of selection and evolution: the conjecture of the theory is that high Assembly configurations of matter (with abundant, large assembly index objects) only occur via evolution and selection. Note in Eq. 1 if *n*_*i*_ = 1, then the contribution of object *i* to the total value of *A* is 0. A recent hypothesized falsification of AT did not address the conceptual foundations of the theory accurately^87^, because it applied the theory to idealized mathematical representations of unit cells, where in real materials, these are not possible to be observed in identical copies due to defects^77^.

## Discussion

In a recent discussion of AT, it was stated that AT cannot account for unanticipated biases, e.g., that AT cannot distinguish whether the bias is natural selection or something else that might favor production of an object given the “rule-based world” that AT is modeling^25^. However, this criticism is made without looking at the empirical data and seemingly without interacting with the ontology of the assembly index. AT is a link between the abiotic world and the biological world and shows that a much more generalized mechanism of selection must have acted before genetic selection appeared. That is, AT makes explicit that selection is a more general phenomenon than that found just in biology. Our central claim is that the combinatorial space is so large, that some configurations simply cannot exist without “systematic bias” stored in a generic memory, what we call “selection,” which will include modern genetic evolution, but also many other more general modes of selection. So far, the central claim holds up to empirical tests, as presented previously^11^ and those are not explored in criticisms of AT. Also, critiques do not provide empirical evidence to support the argument that the mundane deviations from expected outcomes they cite would falsify the central hypotheses of AT by producing arbitrarily high Assembly configurations of matter in abiotic settings. To date, there exists no empirical evidence that natural environments, outside of the processes of life, can produce objects of arbitrarily high complexity.

An interesting critique is related to how AT does not explicitly address function^25^. However, a goal of AT is to measure evolved complexity without referencing function explicitly. This is intentional, as it avoids the issue of predefining all possible functions of a given object where there will always be some unanticipated context in which the object might acquire a new function^88^. There is some debate as to whether a relevant measure of complexity should include explicit reference to context or be context-free. For example, the environmental context or functional context might inform how one quantifies complexity^89,90^. This point is similar to early debates on measuring temperature, where standardization of measurement was challenged by different contexts for experimental protocols, such as material structure of the vessel, pressure, purity of sample, etc^38^. However, ultimately, a fixed temperature scale was developed, which could provide standardized comparison across all these different experimental protocols. It is our contention that the most significant advances in the science of measuring complexity will come from similarly standardized measurements, and assembly theory has been developed with this goal in mind. It is important to note that there are two types of context dependence: The first is whether a mathematical or physical measure is context-dependent. The second is whether the outcomes, possibilities, states, etc. of a particular physical process are context-dependent, even if the measure describing those features is not, which is the case for temperature. Temperature is a universal measure that we use in diverse situations ranging from the surface temperature of the sun to the internal temperature of the ocean: the scale we use to measure these temperatures is the same and fixed across all environments, but the specific values we observe are context-dependent. The assembly index as a measure is context-free; it is like temperature. However, the values achievable or observed are context-dependent. Planetary contexts will determine how far selection can go in generating higher AI.

Another reason to focus on constructability rather than functionality is that it is unclear if the possible uses of a complex object are finite or (at least asymptotically) infinite^91^. If one has *N* objects, there are potentially ~N^N^ possible combinatorial interactions, meaning the number of possible interactions (functions) trends super-exponentially faster toward infinity than the number of objects does as the system size grows. However, while the number of functions an object can perform is not pre-statable^92^, the number of functions that could have constructed any observed object is always finite and well-defined. This is exactly what underlies the definition of the assembly index and the assembly space: AT explains how an object found in high copy number is constructed by functions, rather than what function it has, specifically because this is finite and measurable, as opposed to undefined. As an explicit example of the utility of this approach, one can consider the long-standing paradoxical question of how to tell if a given sequence of DNA is “functional” or not, without referring to the context of the cell^93^. AT would instead ask whether the abundant DNA found in the cell was evidence of selection or not. A similar approach is taken in population genetics, where functionality of a sequence can be detected by finding many copies of it across the genome—we can detect functional jumping elements by finding those that have a large number of copies, vs. mutated non-functional ones that will have only a single copy. We can also find functional elements across different species by finding those that appear preserved across the species. Thus, within an evolutionary context, copy number is an indicator of function. In all these cases, the copying of the genome itself is a given—non-functional parts of the genome will replicate just as well as functional parts across generations. But where they fail is in the first to jump across the genome, and in the second to resist mutations through selection over the population.

Well-developed theories often lead to counterintuitive reformulations of our understanding of phenomenon that depart from our colloquial understanding of them, reframing the question being asked. It may be the case that a formal theory of life will require abandoning our biases about the role and nature of function, to learn something deeper by building abstractions that tie more closely to what empirical data we can objectively measure. Whether AT will continue to pass tests of its empirical validation or be falsified will be determined by future work of the scientific community. But one should not claim a theory invalid because it does not conform to prior expectations of what explanations we should find. Instead, we must always be open as scientists to theories we can rigorously test, and the explanations they provide.

We have shown that the assembly index is not identical to measures of computational complexity and compression, and we have discussed how the phenomenology of assembly theory as a theory of the physical world rooted in measurement distinguishes it from computational complexity theory. Assembly theory was designed to explore how the evolution of evolution was possible before formalized genetics. That is, AT aims to elucidate how selection produces the machinery of modern genetic evolution. One of the challenges with traditional computational complexity theory as an approach to physics and biology is that it has been applied to quantify the amount of complexity found in the natural world using an ontology that may not be meaningful in this context, using metrics that cannot be meaningfully mapped directly to physical measurement. Whereas the other measures described herein are in some cases undecidable, and in all cases dependent on choices of data representation, AT provides a formalization constructed from the physics that generated the object with a grounding in metrology. Furthermore, AT makes predictions falsifiable through empirical observations. One prediction is that objects experimentally inferred to have high assembly index and copy number are uniquely associated with life. Of course, falsifiability is not unique to AT as a scientific theory. What is unique is the ability to use readily available biochemistry lab equipment to obtain fragmentation patterns and copy number data that map to the AT framework, making it the first theory tailored to life detection that is amenable to experimental validation.

## Methods

Methods for computing the assembly indices of strings were implemented using algorithms developed in Seet et al.^70^. The assembly indices of strings were compared to Lempel–Ziv–Welch (LZW) compression and Huffman coding using standard algorithms for implementing these compression algorithms. For comparison across a larger sample, values of assembly index and LZW were computed across all 1,681,680 permutations of “aaaaaabbbbbbcccc”. For a proof of assembly index calculations being NP-hard, we performed a Karp reduction of the vertex cover algorithm to the calculation of assembly index (see supplement).

## Supplementary information

Supplementary Information

## Data Availability

All data is available in the SI.
