# Supplementary material for: Assembly theory and its relationship with computational complexity
Source: Npj Complex. 2025 Sep 3;2(1):27. doi: 10.1038/s44260-025-00049-9 (PMC12408342; doi:10.1038/s44260-025-00049-9)
Supplement: Supplementary file 1 — Supplementary Information [file 44260_2025_49_MOESM1_ESM.pdf]

Assembly Theory and its Relationship with Computational Complexity (Supplementary Material)

Christopher P. Kempes, Michael Lachmann, Andrew Iannaccone, G. Matthew Fricke, M. Redwan Chowdhury, Sara I. Walker, Leroy Cronin

August 11, 2025

1 Introduction

We prove that the assembly index problem (AssemblyIndex) is **NP-Complete**[1] and is therefore computable but intractable. Large instances of problems in this class require heuristic approaches that provide approximate solutions. In this proof, we rigorously define AssemblyIndex following Marshall et al. [10], where assembly theory was originally introduced, as this is critical for understanding its mathematical properties.

Computer scientists classify problems into complexity classes based on the computational resources required to solve them (see Arora and Barak [1] for a thorough discussion). Some important classes include **P**, **NP**, **NP-Complete**, and **NP-Hard**, which describe the *time* complexity of problems in each class. Problems in **P** can be solved by a deterministic Turing Machine (DTM) [14][2] in time bounded by a polynomial function of the input size. Problems in **P** are considered tractable. Solutions to problems in **NP** can be found in exponential time by a DTM,[3] while **NP-Hard** problems take at least as much time to solve as any problem in **NP** and are considered intractable. The class **NP-Complete**, to which we show AssemblyIndex belongs, contains problems that require no more than exponential time, since $\mathbf{NP} \subseteq \mathbf{EXP}$,[4] but strictly more than polynomial time, assuming $\mathbf{P} \neq \mathbf{NP}$.[5]

That AssemblyIndex is **NP-Complete** sets it apart from superficially similar problem classes, such as those arising from Lempel-Ziv (LZ) [16] compression algorithms, Huffman coding [7], and Kolmogorov complexity (KC) [9]. Both LZ and Huffman coding are in **P**, whereas KC is uncomputable and thus lies outside any complexity class.

LZ algorithms prioritize efficiency over optimality, while KC is optimal but cannot be computed for all inputs. Even computable subsets of KC that extend beyond trivial or structured cases, such as MINcKT-SAT, are **NP-hard** [6]. Although LZ algorithms can efficiently estimate an upper bound on KC, the tightness of the bound varies significantly and is problem-dependent [15].

Some polynomial-time algorithms have extremely large exponents (e.g., the Picture Hanging Problem is in **P** but runs in $O(n^{43737})$ [3]), while some superpolynomial problems grow relatively slowly (e.g., Planar Hamiltonian Circuit is **NP-Hard** but has time complexity $O(C^{\sqrt{n}})$ [5]). However, for sufficiently large inputs, the worst-case running time of **NP-Hard** problems is strictly greater than that of problems in **P**.

This distinction has practical implications for computing AssemblyIndex. As problem sizes grow, solving them becomes computationally infeasible, forcing solvers to rely on heuristics that do not guarantee exact solutions. The threshold for infeasibility is context-dependent; for instance, W. Cook, D. Espinoza, M. Goycoolea, and K. Helsgaun exactly solved the **NP-complete** Euclidean Travelling Salesperson Problem (TSP) for 49,687 British pubs with 10^{211761} possible tours [4]. They used linear and parallel programming to complete the computation using 250 years of CPU time.

Clever algorithmic strategies and increased computational resources can handle large instances, but for **NP-complete** problems, the computational effort scales superpolynomially, making exact solutions in-

[1] A problem is an *element* of a complexity class (e.g., AssemblyIndex $\in$ **NP-Hard**). However, by convention, we will often write AssemblyIndex *is* **NP-Hard**.

[2] DTMs model physical computation.

[3] That is, in non-deterministic polynomial (NP) time by a non-deterministic Turing Machine (NTM). NTMs are theoretical models of computation.

[4] **EXP** is the class of decision problems solvable by a DTM in time $O(2^{p(n)})$, where $p(n)$ is a polynomial function of, n, the problem size.

[5] This is the famous $\mathbf{P} \neq \mathbf{NP}$ conjecture. If $\mathbf{P} = \mathbf{NP}$, the consequences would be profound; for example, all provable mathematical statements of length $< N$ could be found in time polynomial in N.

feasible beyond a certain size. While we do not explore the parallelizability of AssemblyIndex or the upper bounds on practical problem sizes here, these factors will ultimately determine computational feasibility.

On the other hand, AssemblyIndex can be solved in at most exponential time, setting it apart from harder problems such as the Halting Problem, which is undecidable, and KC, which is uncomputable.

2 Proof of NP-Completeness

We prove that the AssemblyIndex problem is a member of the classes **NP** and **NP-Hard**, and is therefore **NP-Complete**. Our strategy is to demonstrate that AssemblyIndex is **NP-Hard** by providing a Karp reduction from the Vertex Cover Problem (VertexCover) to the computation of AssemblyIndex. VertexCover is known to be **NP-Hard** [8]. That is, we show that any instance of VertexCover can be solved by reformulating it as an AssemblyIndex problem, and that this transformation can be performed in polynomial time. Computing the AssemblyIndex is therefore at least as hard as solving VertexCover, since VertexCover instances form a subset of AssemblyIndex problems. To establish that AssemblyIndex is not only **NP-Hard** but also **NP-Complete**, we prove that AssemblyIndex is a member of **NP**.

Definition 1 (Karp Reduction)**.** *Given decision problems A and B, A is Karp reducible to B if there is a function f such that 1) for every instance* x *of problem* A, x *is a* yes *instance of* A *if and only if* $\mathrm{f(x)}$ *is a* yes *instance of B and 2) f can be computed in polynomial time [8]. We denote the Karp reduction of problem A to problem B with* $\mathrm{A} \leq_m^p \mathrm{B}$.

Definition 2 (NP-Complete)**.** *For problem* $\mathrm{p}, \mathrm{p} \in$ ***NP-Complete*** *if* $\mathrm{p} \in \boldsymbol{NP} \wedge \forall \mathrm{x} \in \boldsymbol{NP}, \mathrm{x} \leq_m^p \mathrm{p}$. *That is, for a problem to be in the class of **NP-Complete** problems it must be in the class of **NP** problems but also as hard as every other **NP-Complete** problem [2]. Equivalently **NP-Complete** = **NP** ∩ **NP-Hard**.*

Definition 3 (Vertex Cover)**.** *Given a graph* $\mathrm{G} = (\mathrm{V}, \mathrm{E})$*, the vertex cover decision problem asks whether there is a subset* $\mathrm{C} \subset \mathrm{V}$ *with* $|\mathrm{C}| \leq \mathrm{k}$ *such that every edge in* E *includes at least one vertex in* C *[8].*

The proof is divided into four sections: In Section 3 we define the terms used throughout the proof, in Section 4 we prove that AssemblyIndex is in **NP**, and that it is **NP-Hard** in Section 5. Finally we bring everything together in Section 6 to prove that AssemblyIndex is **NP-Complete**.

3 Formalising Assembly Spaces

We use the AssemblyIndex definitions from Marshall et al. [10] where the problem was originally introduced, but reformulate them for clarity in this context. For example, we use graph theory terms rather those of path algebra.[6] We also introduce some novel related definitions and notation, and we also restate some definitions from graph theory for the convenience of the reader.

To mathematically specify a set of assembly objects and the ways in which they can be assembled, we use a directed graph with labeled edges. The vertices of the graph represent the assembly objects, and the directed edges represent allowed assembly operations.

Definition 4 (Edge-labeling Map)**.** *Given a directed graph* Γ *with vertices* $\mathrm{V}(\Gamma)$ *and edges* $\mathrm{E}(\Gamma)$*, an* edge-labeling map *is a function* $\phi : \mathrm{E}(\Gamma) \to \mathrm{V}(\Gamma)$ *that assigns a vertex to every edge of the graph.*

Definition 5 (Edge Bracket Notation)**.** *In the context of a directed graph with vertices* x *and* z*, we use* $\mathrm{e} = [\mathrm{x}, \mathrm{z}]$ *to indicate that* e *is a directed edge from vertex* x *to vertex* z.

Definition 6 (Reachable)**.** *A vertex z in a directed graph* Γ *is **reachable** from vertex* x *if, and only if, there is a path along directed edges in* Γ *from* x *to* z*. Every vertex can also reach itself along a zero length edge, i.e. the relation is reflexive.*

Definition 7 (Source Vertex Set: $\mathrm{Src}(\Gamma)$)**.** *A vertex in a directed graph* Γ *is a **source vertex** in* Γ *if it is reachable only from itself, i.e. it has indegree 0. We use Src*(Γ) *to denote the set of source vertices in* Γ*.*[7]

Definition 8 (Assembly Space)**.** *An **assembly space*** $\Omega = (\Gamma, \phi)$ *is an acyclic directed graph*[8] Γ *together with an edge-labeling map* ϕ *for which:*

1. *Src*(Γ) *is finite and non-empty,*
2. *Every vertex in* Γ *is reachable from a vertex in Src*(Γ)*, and*
3. *If an edge* $\mathrm{e} = [\mathrm{x}, \mathrm{z}] \in \mathrm{E}(\Gamma)$ *and* $\phi(\mathrm{e}) = \mathrm{y}$*, then there exists an edge* $\mathrm{e}' = [\mathrm{y}, \mathrm{z}] \in \mathrm{E}(\Gamma)$ *for which* $\phi(\mathrm{e}') = \mathrm{x}$

This definition is less complex than it may initially appear. The first two criteria hold trivially for any finite, non-empty graph Γ, while the third criterion can be broadly interpreted as stating that objects do not depend on the order in which they were constructed, i.e., assembly operations are commutative. We adopt

[6] For example, we refer to directed graphs rather than quivers and source vertices rather than the minimal set.

[7] Marshall et al. 2019 [10] instead use $\min(\Gamma)$ for the set $\mathrm{Src}(\Gamma)$ and say the vertices in this set are *minimal* in Γ.

[8] Marshall et al. use the equivalent term “quiver”.

this definition for consistency with existing literature. However, since this proof does not consider empty or infinite assembly spaces, we recommend that the reader refer to the more concise Definition 11 for a definition of a *finite* assembly space, which is sufficient for this proof.

Definition 9 (Assembly Object). *If $\Omega = (\Gamma, \phi)$ is an assembly space, we refer to the vertices of the directed graph Γ as its assembly objects, or just its objects. That is, we define*

$$V(\Omega) \triangleq V(\Gamma) \tag{1}$$

The assembly objects of the assembly space Ω are the vertices of its directed graph Γ.

Definition 10 (Assembly Operations). *If $\Omega = (\Gamma, \phi)$ is an assembly space, and there is an edge $\mathrm{e} = [\mathrm{x}, \mathrm{z}] \in \mathrm{E}(\Gamma)$ with $\phi(\mathrm{e}) = \mathrm{y}$, we call this labeled edge an assembly operation (or simply operation) and denote it by*

$$[x + y \to z] \tag{2}$$

Equivalently, we will write that the assembly operation is allowed in Ω, and that this operation assembles z from x and y. We define $E(\Omega)$ as the set of all allowed assembly operations in Ω, so we will also write

$$[x + y \to z] \in E(\Omega) \tag{3}$$

Alternatively, if there is no such edge $\mathrm{e} = [\mathrm{x}, \mathrm{z}] \in \mathrm{E}(\Gamma)$ with $\phi(\mathrm{e}) = \mathrm{y}$, we say that $[x + y \to z]$ is not allowed in Ω and,

$$[x + y \to z] \notin E(\Omega) \tag{4}$$

This allows us to rewrite the third criterion of Definition 8 as:

$$[x + y \to z] \in \mathrm{E}(\Omega) \iff [y + x \to z] \in \mathrm{E}(\Omega) \tag{5}$$

It is possible to further simplify Definition 8 if we are only interested in assembly spaces that have a finite, non-zero number of assembly objects.

Definition 11 (Finite Assembly Space). *A finite assembly space $\Omega = (\Gamma, \phi)$ is a finite, non-empty, acyclic directed graph Γ together with an edge-labeling map ϕ for which:*

$$[x + y \to z] \in E(\Omega) \iff [y + x \to z] \in E(\Omega) \tag{6}$$

Lemma 1. *Every finite assembly space is an assembly space.*

Proof. Let a finite assembly space $\Omega = (\Gamma, \phi)$ be given. The third part of Definition 8 is equivalent to Equation (6), so we only need to verify the first and second parts:

Algorithm 1 Find a path from some source vertex u to a given vertex v.

Require: Directed Acyclic Graph $G = (V, E)$, target vertex v
Ensure: Returns a path from any source vertex u to v, or **nil** if no such path exists

```
function PathToSourceVertex(G, v)
    Input: Directed Acyclic Graph G = (V, E), target vertex v
    Output: A path from any vertex u with only outgoing edges to v, or nil
    visited ← False for all x ∈ V
    function DFS(x)
        visited[x] ← True
        for each y such that [y, x] ∈ E do
            if not visited[y] then
                path ← DFS(y)
                if path ≠ nil then
                    return path + [y]
        if ∀w ∈ V, (w, x) ∉ E then        ▷ Check if x has only outgoing edges
            return [x]
        return nil
    return DFS(v)
```

1. $\mathrm{Src}(\Gamma) \neq \emptyset$ and is finite:
   (a) $\mathrm{Src}(\Gamma)$ is finite because $\mathrm{Src}(\Gamma) \subset V(\Gamma)$, and $V(\Gamma)$ is finite.
   (b) To show that $\mathrm{Src}(\Gamma) \neq \emptyset$: choose any vertex $v \in \Gamma$ and apply Algorithm 1, which returns a path from some source vertex u to v (with the possibility that $u = v$). Note that Algorithm 1 cannot loop indefinitely because the graph is finite and acyclic. Because $u \in \mathrm{Src}(\Gamma)$, $\mathrm{Src}(\Gamma) \neq \emptyset$.
2. For any arbitrary $v \in V(\Gamma)$, Algorithm 1 returns the path from some vertex $u \in \mathrm{Src}(\Gamma)$ to v, so v is reachable from u.

□

Definition 12 (Assembly Subspace). *Given two assembly spaces $\Omega = (\Gamma, \phi)$ and $\Omega' = (\Gamma', \phi')$, we say that Ω' is a subspace of Ω if Γ' is a subgraph of Γ and the edge-labeling functions ϕ and ϕ' are consistent. That is, if*

1. $V(\Gamma') \subset V(\Gamma)$
2. $E(\Gamma') \subset E(\Gamma)$
3. $\phi'(e) = \phi(e) \forall e \in E(\Gamma')$.

The assembly subspace relationship is denoted $\Omega' \subset \Omega$.

Definition 13 (Basis). *An assembly object x in an assembly space $\Omega = (\Gamma, \phi)$ is a* ***basic object*** *in Ω, if $x \in Src(\Gamma)$. We call the set of all basic objects the* ***basis*** *of the assembly space, and we denote it B_Ω. That*

is,

$$B_\Omega = Src(\Gamma) \quad (7)$$

Definition 14 (Composite Object)**.** x *is a composite object in an assembly space* Ω *if* $\mathrm{x} \in V(\Omega)$ *and* $\mathrm{x} \notin B_\Omega$*.*

Note that an object x in an assembly space Ω is a composite object if and only if there is an allowed assembly operation in $E(\Omega)$ that assembles it. [9]

Definition 15 (Rooted Subspace)**.** *We say that a subspace* Ω' *of an assembly space* Ω *is* ***rooted in*** Ω *if* $B_{\Omega'} \subset B_\Omega$*. We also say that* Ω' *is a* ***rooted subspace*** *of* Ω*.*

Conceptually, a rooted subspace is one in which every object can be assembled from the basic objects of the original space.

4 Assembly Index Decision Problem

Definition 16 (AssemblyIndex)**.** *For an assembly object x in an assembly space* Ω*, we say the* **Assembly Index** ***of x in*** Ω *is the minimum number of composite objects in any rooted assembly subspace of* Ω *that contains x. We denote this number* a_x*.*

Complexity classes are defined in terms of decision problems rather than optimisation problems. For the remainder of this proof AssemblyIndex refers to the decision problem unless otherwise specified.

Definition 17 (The AssemblyIndex Problem)**.** *The* **AssemblyIndex decision *problem*** *is: Given an assembly space* $\Omega = (\Gamma, \phi)$ *containing an object* x*, is there a rooted subspace of* Ω *that contains* x *and has no more than* k *composite objects? The input size of the problem is* $N = |\Gamma|$*.*

The *optimisation* version of the problem asks instead for the minimum number of composite objects in any rooted subspace of Ω that contains x. This optimisation can be solved by calling the decision version in Definition 17 as a subroutine a polynomial number of times. For example, by using an $\mathrm{O}(n)$ linear search for a k where the answer is *yes*. Therefore, showing that the decision problem is **NP** and **NP-Hard** classifies the optimisation problem as well.

Definition 17 is the general version of the AssemblyIndex problem– one that is relevant to any type of assembly object: molecules, 2D pixel art, strings, etc. We can also define more limited versions of the problem that are restricted to these specific domains.

Because there has been particular interest in Assembly Theory applied specifically to string objects, we will define the StringAssemblyIndex problem in Section 5.5.1, and we will show that both the general problem and the restricted string version are both **NP-Complete**.

Lemma 2 (AssemblyIndex is in **NP**)**.** *A proposed solution to an* AssemblyIndex *decision problem (certificate) can be checked in polynomial time.*

Proof. Let an assembly space $\Omega = (\Gamma, \phi)$ be given, along with an assembly object x, an integer k, and certificate $\Omega' = (\Gamma', \phi')$. We will show that this candidate solution can be checked in $\mathrm{O}(N^2)$ time, where $N = |\Gamma|$ is the input size of the AssemblyIndex problem.

First, we must check whether Ω' is an assembly space at all. We will presume that the directed graph Γ' is indeed finite, and we can check that it is non-empty in $\mathrm{O}(1)$ time. To check whether Γ' is acyclic, we perform a depth-first search (DFS) of Γ' starting from each of its vertices in turn. If such a search ever reaches a vertex that is already on the recursion stack (a vertex already visited along the current DFS route), then we have found a cycle in Γ', and the certificate fails. If this never happens, then our $|V(\Gamma)|$ depth-first searches will complete in $\mathrm{O}(|\Gamma|^2)$=$\mathrm{O}(N^2)$ time, since a single DFS on an acyclic graph Γ can be performed in $\mathrm{O}(|\Gamma|)$ time. For Ω' to satisfy the definition of a finite assembly space (Definition 11), it remains only to verify that

$$[x + y \to z] \in E(\Omega) \iff [y + x \to z] \in E(\Omega). \quad (8)$$

To do so, we can perform a nested iteration over the edges of Γ'. For each edge $e = [x, z] \in E(\Gamma')$ with $\phi'(e) = y$, we search for an edge $e' = [y, z] \in E(\Gamma')$ with $\phi'(e') = x$. If we fail to find any such edge e', then the certificate fails. If not, then this nested iteration will require $\mathrm{O}(N^2)$ time, and we will know that Ω' is an assembly space.

Next, we will check that $V(\Gamma') \subset V(\Gamma)$, that $E(\Gamma') \subset E(\Gamma)$, and that ϕ and ϕ' label the shared edges consistently. Each check can be done by nested iteration in $\mathrm{O}(N^2)$ time. If all three checks pass, we will know that $\Omega' \subset \Omega$.

Next, we will determine $\mathrm{Src}(\Gamma)$. For each vertex $v \in V(\Gamma)$, we search for an edge in $E(\Gamma)$ whose terminal vertex is v. If no such edge is found, then v is reachable only from itself, so $v \in \mathrm{Src}(\Gamma)$. Repeating this for each vertex will fully determine $\mathrm{Src}(\Gamma) = B_\Omega$ in $\mathrm{O}(N^2)$ time. We can then do the same for $\mathrm{Src}(\Gamma') = B_{\Omega'}$. We then compare the two sets to determine whether $B_{\Omega'} \subset B_\Omega$, which can be done by nested iteration in $\mathrm{O}(N^2)$ time. At this point, we will know whether Γ' is rooted in Γ.

[9] In Marshal's et al. [10] terminology "augmented cardinality" is equivalent to composite object

All that remains is to check whether $x \in V(\Omega')$ and to calculate $|V(\Omega')| - |B_{\Omega'}|$, the number of composite objects in Ω', and compare it to k. At this point, we will have fully checked the certificate in $O(N^2)$ time. □

We now know that the AssemblyIndex problem is in NP. We will prove that it is also **NP-Hard** indirectly, by showing there is a subclass of AssemblyIndex problems that is **NP-Hard**.

5 AssemblyIndex is NP-Hard

We define StringAssemblyIndex to be a subclass of AssemblyIndex problems in which all of the assembly objects are strings, and all of the assembly operations are *commutative* string concatenation. By proving that this subclass is **NP-Hard** we also prove that the superclass is **NP-Hard**.

5.1 The String Assembly Index Decision Problem

Definition 18 (String Product Notation). *When x and y are strings, we use* xy *to denote their concatenation. We use power and product notation similarly, e.g.*

$$\prod_{i=1}^{2} x^i y = (xy)(xxy) = xyxxy \tag{9}$$

Definition 19 (String Assembly Space). *We say an assembly space Ω is a string assembly space if all of assembly objects in* $V(\Omega)$ *are strings, and if*

$$[x + y \to z] \in E(\Omega) \implies z = xy \vee z = yx. \tag{10}$$

That is, if any two objects $x, y \in V(\Omega)$ can be assembled, then the object they assemble must be one of their concatenations, either xy or yx.

Commutativity is a consequence of Definition 8 part 3 and Definition 11. This distinguishes the string assembly operation from standard string concatenation, where $[x + y \to xy]$ and $[y + x \to yx]$. In particular, $[x + y \to xy] \in E(\Omega)$ does not imply $[y + x \to yx] \in E(\Omega)$, instead we have $[y + x \to xy] \in E(\Omega)$. This is not a consequence of how we chose to define the string assembly space for the purposes of this proof, it is a fundamental property of all assembly spaces.

Additionally, Definition 19 does not guarantee that all possible string concatenations are valid assembly operations in Ω. Even if two strings exist in Ω, their concatenation is not necessarily an assembly object. Whether a concatenation is permitted depends on the presence of a corresponding edge in the assembly space's directed graph. In particular:

$$x, y \in V(\Omega) \not\Rightarrow\; xy \in V(\Omega) \tag{11}$$

$$x, y, xy \in V(\Omega) \not\Rightarrow\; [x + y \to xy] \in E(\Omega) \tag{12}$$

$$[x + y \to xy] \in E(\Omega) \tag{13}$$

$$\not\Rightarrow\; [y + x \to yx] \in E(\Omega) \tag{14}$$

Definition 20 (StringAssemblyIndex Problem). *We define the* **StringAssemblyIndex** ***decision problem*** *to be: Given a string assembly space Ω containing a string* x*, is there a rooted subspace of Ω that contains* x *and has no more than* k *composite objects?*

StringAssemblyIndex is a restricted case of AssemblyIndex, so showing that it is **NP-Hard** immediately implies that the general problem is also **NP-Hard**.

5.2 StringAssemblyIndex is NP-Hard

Lemma 3 (StringAssemblyIndex is **NP-Hard**). *Any decision problem in **NP** can be reduced to a* StringAssemblyIndex *decision problem in polynomial time.*

Proof. Following Definition 1 we reduce VertexCover to StringAssemblyIndex, that is, VertexCover$\leq_m^p$StringAssemblyIndex. To do this we show that given any VertexCover problem we can solve it by reduction to an instance of StringAssemblyIndex in a polynomial number of steps. This is accomplished by defining a string assembly space $\Omega = (\Gamma, \phi)$, a string x, and an integer k' so that the resulting StringAssemblyIndex decision problem will always have the same answer as the VertexCover decision problem.

The proof of Lemma 3 proceeds in Sections 5.2.1 to 5.2.6. In Section 5.2.1 we begin the Karp reduction by showing how to construct an assembly space from a vertex cover input graph. In Section 5.2.2 we build intuition around the reduction and discuss related work involving the external pointer method (EPM) [12]. In Sections 5.2.3 and 5.2.4 we show that the reduction meets the certificate requirement from Definition 1, i.e. that we have a a function f for which certificates in StringAssemblyIndex are *yes* instances if and only if the corresponding VertexCover certificate is a *yes* instance. In Section 5.2.5 we show that f takes $O(n^2)$ time to compute and is therefore a valid Karp reduction. Section 5.2.6 completes the proof.

5.2.1 Construct an Assembly Space from a Graph

Before we begin our construction of the assembly space Ω, we enumerate the vertices of the graph $G = (V, E)$,

$$V = \{v_i \mid i = 1 \ldots |V|\} \tag{15}$$

We also enumerate the edges of G, along with their starting and terminal vertices,

$$E = \{e_j = [v_{s_j}, v_{t_j}] \mid j = 1 \ldots |E|\} \tag{16}$$

Note that a single vertex can have multiple identifications within this enumeration system. If the jth edge starts at the mth vertex and terminates at the nth vertex, then we could write $e_j = [v_{s_j}, v_{t_j}] = [v_m, v_n]$.

We next choose a distinct single-character string c_i to correspond to each enumerated vertex $v_i \in V$. We will also define a special single-character string # that is distinct from every other single-character string. To keep the alphabet finite, we encode each vertex as a single k-bit character, with $k = \lceil \log_2 |V| \rceil$, yielding $\Sigma = \{0, 1, \#\}$. Finally, we will use c_{s_j} and c_{t_j} to refer to the single-character strings that correspond to the vertices of each enumerated edge $[v_{s_j}, v_{t_j}]$.

We now begin our construction of the assembly space $\Omega = (\Gamma, \phi)$ by listing all of the (string) assembly objects in $V(\Omega)$, which will be:

1. All of the single-character strings we've just defined:
$$\{\#\} \cup \{c_i \mid v_i \in V\} \tag{17}$$
2. Three composite strings for every vertex v_i:[10]
$$\{\#c_i,\ c_i\#,\ \#c_i\# \mid v_i \in V\} \tag{18}$$
3. One composite string for each edge $e_j = [v_{s_j}, v_{t_j}]$:
$$\{\#c_{s_j}\#c_{t_j}\# \mid e_j = [v_{s_j}, v_{t_j}] \in E\} \tag{19}$$

[10] Throughout the proof of Lemma 3 and particularly in Equations (53) and (54) we use the delimiter # to organise the mapping of substrings to graph elements. This mechanism was first used by Storer and Szymanski [11, 13] to reason about the EPM of string compression. EPM compression replaces substrings in the original data with pointers to identical substrings already processed and stored in a dictionary. The substrings in the dictionary can also contain pointers to other dictionary substrings. The dictionary is analogous to an assembly space, the string to be recovered is like the target object, and the size of the "best" dictionary is reminiscent of the assembly index. The runtime complexity of EPM is $O(n \log n)$ but Storer and Szymanski proved that determining the best dictionary, i.e. one that guarantees optimal compression, is **NP-complete**. Similarly, optimal factorization (minimal phrase substitution) for Lempel-Ziv LZ77 [16] is also **NP-Complete** [11].

4. A sequence of $|V|$ composite strings, formed by starting with # and appending the $\#c_i$ strings one at a time:
$$S_0 = \# \tag{20}$$
$$S_i = (S_{i-1})(\#c_i) \qquad \forall v_i \in V \tag{21}$$
5. Another $|V|$ strings formed by further appending the $c_i\#$ strings to the final string in the previous sequence:
$$S_{|V|+1} = (S_{|V|})(c_1\#) \tag{22}$$
$$S_{|V|+i} = (S_{|V|+i-1})(c_i\#) \qquad \forall v_i \in V \tag{23}$$
6. Another $|E|$ strings formed by further appending the $\#c_{s_j}\#c_{t_j}\#$ strings:
$$S_{2|V|+1} = (S_{2|V|})(\#c_{s_1}\#c_{t_1}\#) \tag{24}$$
$$S_{2|V|+j} = (S_{2|V|+j-1})(\#c_{s_j}\#c_{t_j}\#) \quad \forall e_j \in E \tag{25}$$

The complete set of strings in Ω is thus:

$$V(\Omega) = \{\#\} \cup \{c_i, c_i\#, \#c_i, \#c_i\# \mid 1 \le i \le |V|\} \tag{26}$$
$$\cup \{\#c_{s_j}\#c_{t_j}\# \mid 1 \le j \le |E|\} \tag{27}$$
$$\cup \{S_l \mid 1 \le l \le 2|V| + |E|\} \tag{28}$$

and $\{S_l\}$ is a sequence of $2|V|+|E|+1$ strings, including, for example:

$$S_0 = \# \tag{29}$$
$$S_1 = \#\#c_i \tag{30}$$
$$S_{|V|} = \# \left(\prod_{i=1}^{|V|} \#c_i \right) \tag{31}$$
$$S_{2|V|} = \# \left(\prod_{i=1}^{|V|} \#c_i \right) \left(\prod_{i=1}^{|V|} c_i\# \right) \tag{32}$$
$$S_{2|V|+|E|} = \# \left(\prod_{i=1}^{|V|} \#c_i \right) \left(\prod_{i=1}^{|V|} c_i\# \right) \left(\prod_{j=1}^{|E|} \#c_{s_j}\#c_{t_j}\# \right) \tag{33}$$

Having listed the string assembly objects of $V(\Omega)$, we complete our specification of the assembly space Ω by listing its allowed assembly operations, $E(\Omega)$, which will be the following:[11]

$$[c_i + \# \to c_i\#] \qquad \forall v_i \in V \tag{38}$$
$$[\# + c_i \to \#c_i] \qquad \forall v_i \in V \tag{39}$$
$$[\#c_i + \# \to \#c_i\#] \qquad \forall v_i \in V \tag{40}$$

[11] The attentive reader may notice that Equations (38) and (39) together seem to imply that our assembly operations are non-commutative after all. This is only because we have left the commuted versions of these operations implicit. Equations (38) and (39) actually specify four

$$[\#c_{s_j} + \#c_{t_j}\# \rightarrow \#c_{s_j}\#c_{t_j}\#] \qquad \forall e_j \in E \quad (41)$$
$$[\#c_{s_j}\# + c_{t_j}\# \rightarrow \#c_{s_j}\#c_{t_j}\#] \qquad \forall e_j \in E \quad (42)$$

$$[S_{i-1} + \#c_i \rightarrow S_i] \qquad \forall v_i \in V \quad (43)$$
$$[S_{|V|+i-1} + c_i\# \rightarrow S_{|V|+i}] \qquad \forall v_i \in V \quad (44)$$
$$[S_{2|V|+j-1} + \#c_{s_j}\#c_{t_j}\# \rightarrow S_{2|V|+j}] \qquad \forall e_j \in E \quad (45)$$

This completes our definition of the string assembly space Ω, though it remains to be proven that Ω is indeed a string assembly space. We also define the unlabeled version of the graph Ω as Γ and use the labels to construct an edge-labeling map ϕ, so that $\Omega = (\Gamma, \phi)$.

To prove that $\Omega = (\Gamma, \phi)$ meets the definition of a finite assembly space, first note that the directed graph Γ is finite and non-empty. Next, let an arbitrary edge $[x, z] \in E(\Gamma)$ be given. To exist in the directed graph Γ, this edge must correspond to one of the allowed assembly operations we specified, i.e. $[x + y \rightarrow z] \in E(\Omega)$ for some $y \in V(\Omega)$. For every such assembly operation we defined, z is a string that is strictly longer than the string x. This means that the directed edge $[x, z]$ terminates at a string that is strictly longer than the string from which it began. Because this is true of every edge in $E(\Gamma)$, there can be no cycles in Γ. We now know Γ is a finite, non-empty, acyclic graph, so it remains only to prove that $[x + y \rightarrow z] \in E(\Omega)$ implies $[y + x \rightarrow z] \in E(\Omega)$, but that property was inherent to our method of specifying the edges and edge-labeling– each allowed assembly operation we specified implicitly specified that the commuted version was allowed as well. Thus, Definition 11 of a finite assembly space is satisfied, so Ω is indeed an assembly space.

To see that Ω is also a string assembly space, note that each of the allowed assembly operations we listed has the form $[x + y \rightarrow xy]$, while the commuted version we left implicit has the form $[y + x \rightarrow xy]$ (or equivalently, $[x + y \rightarrow yx]$). In either case, Equation (10) is satisfied, and so Ω is indeed a string assembly space.

Finally, to complete the construction of our StringAssemblyIndex problem, we must define x and k'. For reasons that will become apparent below, we choose:

$$k' = 4|V| + 2|E| + k, \quad (46)$$

where V and E are from the original vertex cover graph $G = (V, E)$; and for x, we choose the final string in the $\{S_l\}$ sequence:

$$x = S_{2|V|+|E|} \quad (47)$$

In the following sections, we will prove that this StringAssemblyIndex decision problem (Ω, x, k') is logically equivalent to the VertexCover decision problem (G, k) from which it was constructed. We will do so by showing that a certificate for the VertexCover problem can be used to produce a certificate for the StringAssemblyIndex problem, and vice versa.

In the subsections of the proof that follow, we will refer to these problems as *the* StringAssemblyIndex *decision problem* and *the* VertexCover *decision problem*, respectively. For the duration of this proof, when we use any of the generic symbols: $G, V, E, k, \Omega, \Gamma, \phi, \{S_l\}, x, k'$, we will mean the particular values given to those symbols in this section. For example, E will refer to the edges of the vertex cover graph $G = (V, E)$, and k' will refer to the value $4|V| + 2|E| + k$.

5.2.2 Intuition

Informally, we have designed Ω so that assembling the string $x = S_{2|V|+|E|}$ from the basic objects of Ω would require first assembling *almost* every other composite string in $V(\Omega)$. This is due to the way we defined the $\{S_l\}$ sequence, directly requiring the successive concatenation of every string in $V(\Omega)$ of the form $c_i\#$, $\#c_i$, or $\#c_{s_j}\#c_{t_j}\#$ along the path from S_0 to $S_{2|V|+|E|}$.

But we have not similarly made the $\#c_i\#$ strings directly required for the $\{S_l\}$ sequence. They are only indirectly required for their role in assembling the other required strings. Specifically, they are needed only to assemble the $\#c_{s_j}\#c_{t_j}\#$ strings. Critically, a rooted subspace of Ω is capable of assembling all of the $\#c_{s_j}\#c_{t_j}\#$ strings if and only if it contains sufficient $\#c_i\#$ strings to form a vertex cover, that is. enough that

$$C = \{v_i \in V \mid \#c_i\# \in V(\Omega')\} \quad (48)$$

includes at least one vertex in V incident to every edge in E, where $G = (V, E)$ is the graph from the VertexCover problem.[12]

This bijection between vertex covers of G and rooted subspaces of Ω is the core mechanism of this proof. It will allow us to use a solution to the StringAssemblyIndex problem to generate a solution to the Ver-

different assembly operations:

$$[c_i + \# \rightarrow c_i\#] \quad (34)$$
$$[\# + c_i \rightarrow c_i\#] \quad (35)$$
$$[c_i + \# \rightarrow \#c_i] \quad (36)$$
$$[\# + c_i \rightarrow \#c_i] \quad (37)$$

That is to say, the string objects c_i and $\#$ can be combined to assemble *either* $c_i\#$ or $\#c_i$, and their order is never relevant.

[12]This relationship will be demonstrated below. It is not meant to be clear to the reader at this time.

TEXCOVER problem, and vice-versa, and thereby to show the problems are equivalent.

5.2.3 StringAssemblyIndex Certificates Map to VertexCover Certificates

Lemma 4. *Given a certificate for the* StringAssemblyIndex *decision problem, that is, a string assembly space* Ω' *that is a rooted subspace of* Ω*, containing the string* x*, and has* k′ *composite objects. We claim that the set*

$$C = \{v_i \in V \mid \#c_i\# \in V(\Omega')\} \tag{49}$$

is a vertex cover of $G = (V, E)$ *with size* k*, i.e. a certificate for the* VertexCover *decision problem.*

Proof. First, we determine B_Ω, which is simply the set of non-composite strings in $V(\Omega)$ and therefore cannot be assembled by any operation in Ω. By comparing the list of strings in $V(\Omega)$ in Section 5.2.1 to the list of allowed assembly operations in Ω specified by Equations (38) to (45), we see that the only such strings are the single-character strings in $V(\Omega)$. That is:

$$B_\Omega = \{\#\} \cup \{c_i | v_i \in V\} \tag{50}$$

Next, we determine $V(\Omega')$, which contains all of the strings in $V(\Omega)$ except those of the form $\#c_i\#$. We will establish this by considering each type of string in $V(\Omega)$ in turn:

1. Each string in the sequence $\{S_l\}$ must be in Ω'. Conceptually, this is because we've designed Ω so that the only way to assemble $x = S_{2|V|+|E|}$ is to assemble each string in the entire S_l sequence. To show this rigorously, we establish the following induction:
   Suppose $S_l \in V(\Omega')$ for some $l \geq 1$. Note that $S_l \notin B_\Omega$, because it is not one of the single-character strings. Since Ω' is rooted in Ω, $S_l \notin B_{\Omega'}$. S_l is therefore a composite string in Ω', implying that there exists an operation in $E(\Omega')$ that assembles it. That operation must be one of those in Equations (43) and (45), all of which have the form $[S_{l-1} + w \to S_l]$ for some string $w \in V(\Omega)$. For such an operation to be allowed in Ω', is is necessary for $S_{l-1}, w \in V(\Omega')$. Thus,

   $$\forall l \geq 1, S_l \in V(\Omega') \implies S_{l-1} \in V(\Omega') \tag{51}$$

   Starting with the assumption that $x \in V(\Omega')$, i.e., $S_l \in V(\Omega')$ for $l = 2|V| + |E|$, the induction proceeds until reaching the base case $l = 1$, establishing that $S_l \in V(\Omega')\ \forall l \geq 0$.
2. For strings in $V(\Omega)$ of the forms $c_i\#$, $\#c_i$, and $\#c_{s_j}\#c_{t_j}\#$ we proceed with proof by contradiction. From Equations (43) to (45) it is clear that strings with these forms are each required for one of the operations by which the S_l strings are assembled. If any one of them were absent from $V(\Omega')$, then one of those assembly operations would not be allowed in Ω', and thus some S_l would not be a composite object in Ω'. This would mean $S_l \in B_{\Omega'}$, which is false, because it is not one of the single-character strings. By contradiction, all strings of these forms in $V(\Omega)$ must also be present in $V(\Omega')$.
3. If any of the single-character strings, $\#$ and $\{c_i\}$ were absent from $V(\Omega')$, then the operation that assembles some string of the form $c_i\#$ or $\#c_i$ would not be allowed in Ω', and one of those strings would instead be in $B_{\Omega'}$. As in the previous argument, this would yield a contradiction, so $\#$ and all of the strings c_i must be in Ω'.
4. Finally, consider the strings of the form $\#c_i\#$. We cannot establish exactly which of these strings are in Ω', but we *can* establish that the vertices that they correspond to comprise a vertex cover in G. First we define C,

   $$C \triangleq \{v_i \in V \mid \#c_i\# \in V(\Omega')\}\,. \tag{52}$$

   Now let an arbitrary edge $e_j = [v_{s_j}, v_{t_j}] \in E$ be given. We know that $\#c_{s_j}\#c_{t_j}\# \notin B_{\Omega'}$, so $\#c_{s_j}\#c_{t_j}\#$ must be a composite string in Ω'. Of the two assembly operations allowed in Ω that can assemble $\#c_{s_j}\#c_{t_j}\#$, at least one must also be allowed in Ω' as well. Either,

   $$\left[\#c_{s_j} + \#c_{t_j}\# \to \#c_{s_j}\#c_{t_j}\#\right] \in E(\Omega') \tag{53}$$

   or

   $$\left[\#c_{s_j}\# + c_{t_j}\# \to \#c_{s_j}\#c_{t_j}\#\right] \in E(\Omega'). \tag{54}$$

   If the first of these operations is allowed in Ω', then all of the strings involved in it must be in $V(\Omega')$. In particular, $\#c_{t_j}\# \in V(\Omega')$, which would mean $v_{t_j} \in C$. Similarly, if the second operation was allowed in Ω', it would mean $v_{s_j} \in C$. In either case, C contains at least one of the vertices of the edge $e_j = [v_{s_j}, v_{t_j}]$. Because this is true for every edge $e_j \in E$, C is a vertex cover of G.

We will further prove that $|C| = k$. To do so, first note that the steps above have determined which strings are in Ω':

$$\begin{aligned}
V(\Omega') = \{&S_l \mid l = 1 \ldots 2|V| + |E|\} && (55)\\
&\cup \{c_i\#, \#c_i \mid i = 1 \ldots |V|\} && (56)\\
&\cup \{\#c_{s_j}\#c_{t_j}\# \mid j = 1 \ldots |E|\} && (57)\\
&\cup \{\#\} \cup \{c_i \mid i = 1 \ldots |V|\} && (58)\\
&\cup \{\#c_i\# \mid v_i \in C\} && (59)
\end{aligned}$$

Therefore,

$$|V(\Omega')| = 1 + 5|V| + 2|E| + |C| \tag{60}$$

We already established that B_Ω consists of all the single-character strings in $V(\Omega)$, and that these strings are also present in $V(\Omega')$. Each such single-character string is a basic object in Ω', and no other objects can be basic objects in Ω' because Ω' is rooted in Ω. Therefore,

$$B_{\Omega'} = \{\#\} \cup \{c_i | v_i \in V\} \tag{61}$$
$$\therefore |B_{\Omega'}| = 1 + |V| \tag{62}$$

Putting these together yields,

$$|V(\Omega')| - |B_{\Omega'}| = 4|V| + 2|E| + |C| \tag{63}$$

Because we began this section with the assumption that Ω' has k' composite objects, we have

$$k' = 4|V| + 2|E| + |C| \tag{64}$$
$$\therefore 4|V| + 2|E| + k = 4|V| + 2|E| + |C| \tag{65}$$
$$\therefore k = |C| \tag{66}$$

Thus, C is a vertex cover of G with size k, as desired. It is a certificate to the original VertexCover decision problem. □

5.2.4 VertexCover Certificates Map to StringAssemblyIndex Certificates

To complete the bidirectional correspondence in our Karp reduction, we now show that every solution to VertexCover yields a valid solution to StringAssemblyIndex.

Lemma 5. *Given a certificate for the* VertexCover *decision problem, that is, a vertex cover* C *of the graph* G = (V, E) *with* |C| = k*, we can use C to construct a string assembly space $\Omega' = (\Gamma', \phi')$ that is a certificate for the* StringAssemblyIndex *decision problem.*

Proof. Conceptually, the subspace $\Omega' = (\Gamma', \phi')$ we will construct is just $\Omega = (\Gamma, \phi)$ with some of the $\#c_i\#$ strings removed, along with the assembly operations that involve those strings. Formally, we define the vertices of the graph Γ' to be

$$V(\Gamma') = V(\Gamma) \setminus \{\#c_i\# | v_i \in V - C\} \tag{67}$$

For the edges of Γ', we include every edge from Γ that we can. That is, we include every edge from Γ whose vertices and label are all included in Γ':

$$E(\Gamma\prime) = \{e = [s, t] \mid e \in E(\Gamma),\ \phi(e) \in V(\Gamma')\} \tag{68}$$

Finally, we define the edge-labelling map ϕ' to be consistent with ϕ:

$$\phi'(e) = \phi(e) \quad \forall e \in E(\Gamma') \tag{69}$$

Note that this construction implies that Ω' has all the same operations as Ω, unless it is missing one of the strings involved in one of those operations. That is,

$$[x + y \to z] \in E(\Omega') \tag{70}$$
$$\iff [x + y \to z] \in E(\Omega) \wedge \{x, y, z\} \subseteq V(\Omega'). \tag{71}$$

Now that we have defined $\Omega' = (\Gamma', \phi')$, we need to prove that it really is an assembly space. We know the graph Γ' is finite and acyclic because it is a subgraph of Γ, and we know it is non-empty because it contains at least #. If $[x + y \to z] \in E(\Omega')$ then Equation (71) tells us that $x, y, z \in V(\Omega')$ and $[x + y \to z] \in E(\Omega)$. Because Ω is an assembly space, the commuted version of this operation is also allowed, i.e. $[y + x \to z] \in E(\Omega)$. Now applying Equation (71) again tells us that $[y + x \to z] \in E(\Omega')$ as well, so Ω' meets the definition of a finite assembly space. To show that it is specifically a string assembly space, note that all of its assembly objects are strings, and that $E(\Omega') \subset E(\Omega)$. Because the assembly operations in $E(\Omega)$ obey Equation (10), those in $E(\Omega')$ do as well, and so Definition 19 is satisfied.

Because the graph Γ' is a subgraph of Γ and the edge-labeling functions ϕ and ϕ' are consistent, Ω' is furthermore an assembly subspace of Ω. To show that Ω' is rooted in Ω, we will show that $B_{\Omega'} = B_\Omega$. In the previous section, we found the basis of Ω to be all of its single-character strings. Each of these strings is in Ω' as well by Equation (67), and each is reachable only from itself in Ω'. Each single-character string is therefore a member of $B_{\Omega'}$.

To show that none of the other strings in Ω' can be in $B_{\Omega'}$, we will consider each in turn:

1. Consider the string $c_i\#$ for any $1 \leq i \leq |V|$. By Equation (67), $c_i, \#, c_i\# \in V(\Omega')$, and Equation (38) says $[c_i + \# \to c_i\#] \in E(\Omega)$, so by Equation (71), $[c_i + \# \to c_i\#] \in E(\Omega')$. Since $c_i\#$ is thus a composite object in Ω', $c_i\# \notin B_{\Omega'}$. A symmetric argument tells us that $\#c_i \notin B_{\Omega'}$.
2. For strings of the form $\#c_i\#$ for any $1 \leq i \leq |V|$, if $c_i \notin C$, then by Equation (67), $\#c_i\#$ is not in $V(\Omega')$, and so it cannot be in $B_{\Omega'}$. Alternatively, if $v_i \in C$, then $\#c_i\#$ is in $V(\Omega')$, so because $\#c_i$ and # are also in $V(\Omega')$, the assembly operation

   $$[\#c_i + \# \to \#c_i\#]$$

   that was allowed in Ω (Equation (40)) is also allowed in Ω'. Again, $\#c_i\#$ is reachable from another string in Ω', so it is not in $B_{\Omega'}$.

3. Next, consider the strings $\#c_{s_j}\#c_{t_j}\#$ for any $1 \leq j \leq |E|$. Because C is a vertex cover of the graph $G = (V, E)$ and $[v_{s_j}, v_{t_j}]$ is one of its edges, C must contain at least one of its vertices. If $v_{s_j} \in C$, then $\#c_{s_j}\# \in V(\Omega')$ by Equation (67). Because $c_{t_j}\#, \#c_{s_j}\#c_{t_j}\# \in V(\Omega')$, the assembly operation
$$[\#c_{s_j}\# + c_{t_j}\# \to \#c_{s_j}\#c_{t_j}\#] \tag{72}$$
that was allowed in Ω by Equation (42) is also allowed in Ω', therefore $\#c_{s_j}\#c_{t_j}\# \notin B_{\Omega'}$ by the same argument made above for strings of the form $\#c_i\#$ for any $1 \leq i \leq |V|$. If instead $v_{t_j} \in C$, we follow the same logic to conclude that the assembly operation
$$[\#c_{s_j} + \#c_{t_j}\# \to \#c_{s_j}\#c_{t_j}\#] \tag{73}$$
from Equation (41) is allowed in Ω', and we again conclude that $\#c_{s_j}\#c_{t_j}\# \notin B_{\Omega'}$.
4. Finally, consider the string S_l for any $1 \leq l \leq |V|$. By the same argument given above, the assembly operation
$$[S_{l-1} + \#c_l \to S_l] \tag{74}$$
from Equation (43) must be allowed in Ω', so S_l is reachable from S_{l-1} in Ω', and therefore is not in $B_{\Omega'}$. If $|V| < l \leq 2|V| + |E|$, the argument is identical, but uses either Equation (44) or Equation (45).

The above arguments cover all possible strings in Ω and thus, the only strings that can form the basis of Ω'. We therefore conclude that $B_{\Omega'}$ consists solely of the single-character strings:
$$B_{\Omega'} = \{\#\} \cup \{c_i | i = 1 \ldots |V|\} = B_\Omega$$
We now know that Ω' is a rooted assembly subspace of Ω that contains x. All that remains is to calculate its number of composite objects. The total number of strings in Ω can be determined using the list in Section 5.2.1:
$$|V(\Omega)| = 1 + 6|V| + 2|E| \tag{75}$$
so together with Equation (67), we have
$$|V(\Omega')| = |V(\Omega)| - (|V| - |C|) \tag{76}$$
$$= (1 + 6|V| + 2|E|) - (|V| - |C|) \tag{77}$$
$$= 1 + 5|V| + 2|E| + |C| \tag{78}$$

$$\therefore |V(\Omega')| - |B_{\Omega'}| \tag{79}$$
$$= (1 + 5|V| + 2|E| + |C|) - (1 + |V|) \tag{80}$$
$$= 4|V| + 2|E| + |C| \tag{81}$$
and we began this section with the assumption that $|C| = k$, so we conclude that the number of composite objects in Ω' is
$$|V(\Omega')| - |B_{\Omega'}| = 4|V| + 2|E| + k = k' \tag{82}$$
To conclude, Ω' is a string assembly space that is rooted in Ω, contains x, and has k' composite objects. That is, it serves as a certificate for the StringAssemblyIndex decision problem, as desired. ☐

5.2.5 Polynomial-Time Reduction

By combining the last two subsections, we have established a reduction from an arbitrary VertexCover decision problem to a corresponding StringAssemblyIndex decision problem. This reduction ensures that a certificate for one problem exists if and only if a corresponding certificate exists for the other. Next we demonstrate that the reduction can be performed in polynomial time, as required by Definition 1.

Lemma 6. *The reduction from* VertexCover *decision problems to corresponding* StringAssemblyIndex *decision problems requires* $O(\mathrm{N}^2)$ *time, where* $\mathrm{N} = |\mathrm{V}| + |\mathrm{E}|$ *is the input size of the* VertexCover *problem.*

Proof. We only need to read the specification of the graph G as input and produce the specification of the assembly space $\Omega = (\Gamma, \phi)$, along with x and k'. This can be done in $\mathrm{O}(N^2)$ time by following the steps beginning in Section 5.2.1 to list the string assembly objects in Ω, and then following Equations (38) and (45) to list its allowed assembly operations. The list of allowed assembly operations already constitutes an unambiguous implicit specification of the edges of Ω and its edge-labeling map ϕ, but each assembly operation can also be transformed in an explicit specification of the two corresponding edges and their labels in $\mathrm{O}(1)$ time.

The number of strings in Ω is $1 + 6|V| + 2|E|$, and the number of allowed assembly operations is $5|V| + 2|E|$. The reduction process requires no additional computation that scales with the input size; it is only copying strings, and each string or assembly operation can be written in $\mathrm{O}(1)$ time. So there are $\mathrm{O}(|V| + |E|) = O(N)$ statements to write, each of length $\mathrm{O}(N)$. So reducing the VertexCover decision problem to our StringAssemblyIndex decision problem requires only polynomial time. ☐

5.2.6 StringAssemblyIndex is NP-Hard

Lemmas 4 to 6 demonstrate that an arbitrary VertexCover decision problem can be reduced

to a StringAssemblyIndex decision problem in polynomial-time and therefore by Definition 1 StringAssemblyIndex is **NP-Hard**. □

6 AssemblyIndex is NP-Complete

Completing the proof that both AssemblyIndex problems are **NP-Complete** is now a formality. The remaining steps follow directly from the fact that StringAssemblyIndex problems form a specific subclass of AssemblyIndex problems and that their optimization versions can be solved with only a polynomial number of calls to the decision versions.

Corollary 1 (StringAssemblyIndex is in **NP**)**.** *A proposed solution (certificate) to a* StringAssemblyIndex *decision problem can be checked in polynomial time.*

Proof. A certificate for a StringAssemblyIndex decision problem is also a certificate for a (general) AssemblyIndex decision problem. Lemma 2 tells us that any certificate in the latter class can be checked in polynomial time, so the result follows. □

Corollary 2 (AssemblyIndex is **NP-Hard**)**.** *Any decision problem in **NP** can be reduced to a (general)* AssemblyIndex *decision problem in polynomial time.*

Proof. By Lemma 3, any decision problem in **NP** can be reduced to a StringAssemblyIndex decision problem in polynomial time. Any such problem is also a (general) AssemblyIndex decision problem, so the result follows. □

Theorem 1 (AssemblyIndex is **NP-Complete**)**.** *The* AssemblyIndex *problem is **NP-Complete**.*

Proof. With Definition 2, Lemma 2, and Corollary 2 the result follows. □

Theorem 2 (StringAssemblyIndex is **NP-Complete**)**.** *The* StringAssemblyIndex *problem is **NP-Complete**.*

Proof. Combining Definition 2, Lemma 3, and Corollary 1 completes the proof. □

Acknowledgements

We thank Ulysse Prieto for valuable discussions of string pathway complexity and its relation to EPM.

References

[1] Sanjeev Arora and Boaz Barak. *Computational complexity: a modern approach.* Cambridge University Press, 2009.

[2] Stephen A Cook. The complexity of theorem-proving procedures. In *Proceedings of the third annual ACM symposium on Theory of computing*, pages 151–158, 1971.

[3] Erik D Demaine, Martin L Demaine, Yair N Minsky, Joseph SB Mitchell, Ronald L Rivest, and Mihai Pătraşcu. Picture-hanging puzzles. *Theory of Computing Systems*, 54:531–550, 2014.

[4] Lance Fortnow. Fifty years of p vs. np and the possibility of the impossible. *Communications of the ACM*, 65(1):76–85, 2021.

[5] Michael R Garey, David S. Johnson, and R Endre Tarjan. The planar hamiltonian circuit problem is np-complete. *SIAM Journal on Computing*, 5(4):704–714, 1976.

[6] Shuichi Hirahara. Unexpected hardness results for kolmogorov complexity under uniform reductions. In *Proceedings of the 52nd Annual ACM SIGACT Symposium on Theory of Computing*, pages 1038–1051, 2020.

[7] David A Huffman. A method for the construction of minimum-redundancy codes. *Proceedings of the IRE*, 40(9):1098–1101, 1952.

[8] Richard M. Karp. Reducibility among combinatorial problems. In Raymond E. Miller, James W. Thatcher, and Jean D. Bohlinger, editors, *Complexity of Computer Computations*, pages 85–103. Plenum Press, New York, 1972.

[9] Andrei N Kolmogorov. Three approaches to the quantitative definition ofinformation'. *Problems of information transmission*, 1(1):1–7, 1965.

[10] Stuart M. Marshall, Douglas G. Moore, Alastair R. G. Murray, Sara Imari Walker, and Leroy Cronin. Quantifying the pathways to life using assembly spaces. *CoRR*, abs/1907.04649, 2019.

[11] J Storer. Np-completeness results concerning data compression. *Technical Report 234*, 1977.

[12] James A Storer and Thomas G Szymanski. The macro model for data compression. In *Proceedings of the tenth annual ACM symposium on Theory of computing*, pages 30–39, 1978.

[13] James A Storer and Thomas G Szymanski. Data compression via textual substitution. *Journal of the ACM (JACM)*, 29(4):928–951, 1982.

[14] AM Turing. On computable numbers, with an application to the entscheidungsproblem. *J. of Math*, 58:345–363, 1936.

[15] Hector Zenil. A review of methods for estimating algorithmic complexity: Options, challenges, and new directions. *Entropy*, 22(6):612, 2020.

[16] Jacob Ziv and Abraham Lempel. A universal algorithm for sequential data compression. *IEEE Transactions on information theory*, 23(3):337–343, 1977.
